# Cholesterol-directed nanoparticle assemblies based on single amino acid peptide mutations activate cellular uptake and decrease tumor volume†
†Electronic supplementary information (ESI) available. See DOI: 10.1039/c7sc02616a



**DOI:** 10.1039/c7sc02616a

**Published:** 2017-09-11

**Authors:** Shang Li, Rongfeng Zou, Yaoquan Tu, Junchen Wu, Markita P. Landry

**Affiliations:** a Key Laboratory for Advanced Materials & Institute of Fine Chemicals , School of Chemistry and Molecular Engineering , East China University of Science and Technology , Shanghai 200237 , China . Email: jcwu@ecust.edu.cn; b Department of Chemical and Bio-molecular Engineering , University of California Berkeley , 476 Stanley Hall , Berkeley , California 94720 , USA . Email: landry@berkeley.edu; c California Institute for Quantitative Biosciences (qb3) , University of California-Berkeley , Berkeley , CA 94720 , USA; d Division of Theoretical Chemistry and Biology , School of Biotechnology , KTH Royal Institute of Technology , SE-10691 Stockholm , Sweden

## Abstract

Peptide drugs have been difficult to translate into effective therapies due to their low *in vivo* stability. Here, we report a strategy to develop peptide-based therapeutic nanoparticles by screening a peptide library differing by single-site amino acid mutations of lysine-modified cholesterol. Certain cholesterol-modified peptides are found to promote and stabilize peptide α-helix formation, resulting in selectively cell-permeable peptides. One cholesterol-modified peptide self-assembles into stable nanoparticles with considerable α-helix propensity stabilized by intermolecular van der Waals interactions between inter-peptide cholesterol molecules, and shows 68.3% stability after incubation with serum for 16 h. The nanoparticles in turn interact with cell membrane cholesterols that are disproportionately present in cancer cell membranes, inducing lipid raft-mediated endocytosis and cancer cell death. Our results introduce a strategy to identify peptide nanoparticles that can effectively reduce tumor volumes when administered to in *in vivo* mice models. Our results also provide a simple platform for developing peptide-based anticancer drugs.

## Abstract 2

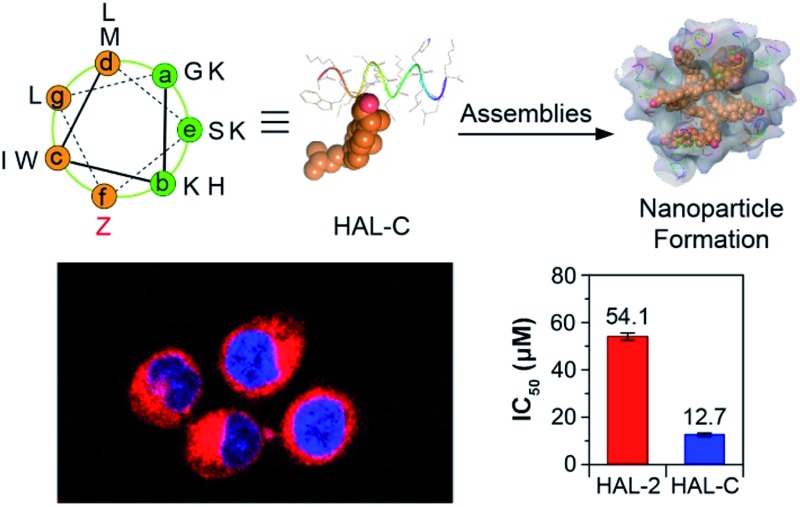
Peptide drugs have been difficult to translate into effective therapies due to their low *in vivo* stability.

## Introduction

*De novo* design of peptide therapeutic agents requires both cell permeability and *in vivo* stability. α-helices are secondary structures found in nearly every native protein, and as peptides, have been shown to effectively internalize into cells.^1^ However, α-helical peptides alone are susceptible to proteolysis, and lack structural stability provided when these domains are found within a protein. As such, three principal challenges currently limit targeted intracellular delivery of anticancer functional peptides to cancer cells and *in vivo*: (I) individual peptides must adopt and maintain a robust α-helical conformation to maintain therapeutic efficacy. (II) Individual peptides must not be susceptible to proteolysis when administered *in vivo*, (III) cancer cell membranes exhibit reduced fluidity due to their disproportionately high cholesterol content, thus anticancer peptides must penetrate through the viscous cholesterol-rich lipid bilayers of cancer cell membranes.^2^ Therefore, if α-helical peptides can be designed at the molecular level to have *in vivo* stability and target internalization through cholesterol-rich cell membranes, α-helical peptides could be used as anticancer drugs.^3^ However, to date, the exact mechanism by which a polypeptide chains fold into a secondary structure remains elusive. Therefore, designing peptide or protein from primary sequence that will adopt a stable α-helical secondary structure, one that is protected against proteolysis under *in vivo* circulation, remains difficult.

Several approaches have been explored to stabilize α-helical therapeutic peptides by covalently linking *i* and either *i* + 4 or *i* + 7 amino-acid residues with amide bonds,^4^ hydrocarbon bonds connected by olefin metathesis,^5^ and triazoles connected between azides and alkynes.^6^ While these methods effectively reduce the conformational flexibility of peptide, covalent linkers require harsh reaction conditions not easily amenable to peptide and protein substrates, and also require the peptide to have two reactive groups in the backbone.^7^

We instead wish to exploit biological self-assembly and non-covalent interactions inherent to protein folding as a design strategy for α-helical peptide therapy development. We seek to maximize the α-helicity and *in vivo* stability by inducing hydrogen bonding, electrostatic interactions, and hydrophobic interactions between peptide units.^8^ Increasing electrostatic interactions between peptides may induce intra-peptide nanoparticle self-assembly, whereby the resulting nanoparticle could protect individual α-helix monomers, much like the stability of α-helices is protected when folded into a larger protein structure. Lastly, introducing cholesterol along the α-helical peptide can favour peptide interaction with the disproportionate number of cholesterol units on cancer cell membranes. For this reason, we choose to leverage the overabundance of cell surface cholesterol on cancer cells to favour interactions between our therapeutic peptides and cancer cells.

We seek to identify cholesterol-modified α-helical peptides that can self-assemble into *in vivo* stable nanoparticles. To this end, we developed a small peptide library (*N* = 4) where each peptide differs by a single-site amino acid mutation of lysine-modified cholesterol. By introducing cholesterol units, we aim to increase the hydrophobic microenvironment of the peptide to (I) stabilize the α-helicity of peptides and (II) induce spontaneous self-assembly of peptides into nanostructures. The stable nanostructures could activate the cell's lipid raft-mediated endocytosis pathway in the cholesterol-rich domain of cancer cell membranes, thereby maximizing interactions between therapeutic peptides and cancer cells.^8^

Based on the above considerations, we chose the base sequence of our peptide as halictines-2 (ref. 9) (HAL-2, Gly–Lys–Trp–Met–Ser–Leu–Leu–Lys–His–Ile–Leu–Lys–NH_2_), from which to introduce cholesterol point mutations. HAL-2 is an antimicrobial peptide, but has low potency against cancer cells.^10^ Thus, we seek to increase HAL-2 anticancer efficacy both *in vitro* and *in vivo* by introducing lysine cholesterol modifications on the base HAL-2 peptide sequence.

## Results and discussion

**Fig. 1:**
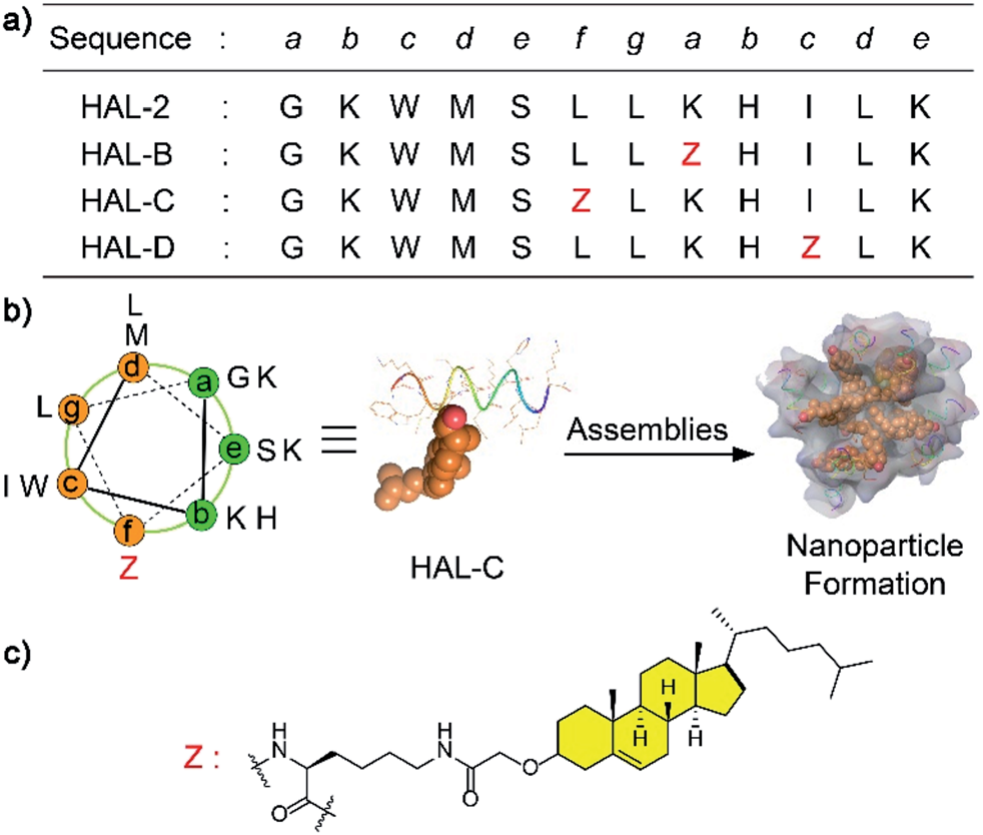
(a) Sequences of HAL-2, HAL-B, HAL-C and HAL-D. Heptad positions are shown in italics. (b) Helical wheel diagram of the HAL-C sequence. Z letter refers to a substituted heptad f position in HAL-2 (orientation as in (a)). Energy-minimized α-helical structure of HAL-C (side view) and the conceptual nanoparticle formed by HAL-C. The gold balls refer to the cholesterol structure. The cartoon helices of HAL-C formed nanoparticles *via* the interaction between cholesterol molecules. (c) A cholesterol structure unit is modified in the lysine side (Z).

Our peptide design is based on the “a–b–c–d–e–f–g” heptad repeat rule, and accompanying folding and self-assembly simulations, where the hydrophobic (c, d, f, g) and hydrophilic (a, b, e) faces of the HAL-2 peptide drive α-helical folding.^11^ We selectively replace one residue, denoted ‘Z’, on either hydrophilic or hydrophobic peptide face to generate three peptides, HAL-B and HAL-C, and HAL-D respectively (Fig. 1), to introduce cholesterol at each site with the intention of understanding the consequences of cholesterol incorporation at these sites, and their subsequent impact on the self-assembly and secondary structure of the resulting peptide nanoparticle. The Fmoc-protected lysine side chain-connected cholesterol unit was synthesized as previously described.^12^ Subsequently, HAL-2, HAL-B, HAL-C and HAL-D were synthesized by Fmoc-based solid-phase synthesis. Amino acids coupled on a Rink amide resin were cleaved from the resin, and protecting groups were removed (Fig. S1–S4, ESI†).

**Fig. 2:**
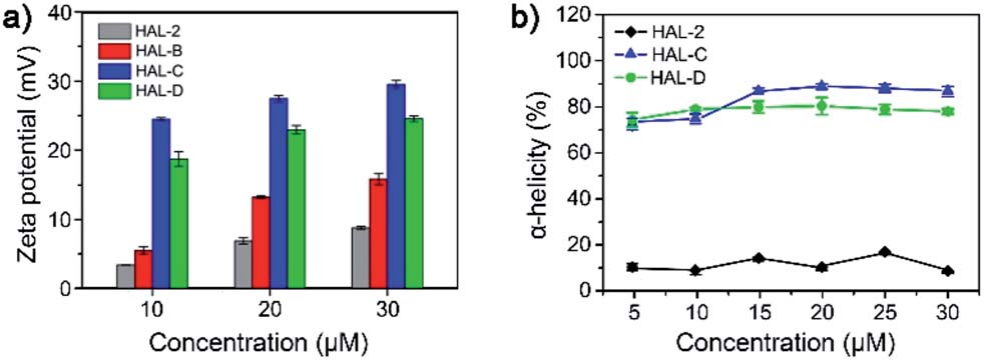
(a) Zeta potentials of HAL-2, HAL-B, HAL-C and HAL-D in TBS (10, 20 and 30 μM, pH 7.4). (b) Helix propensity of HAL-2, HAL-C and HAL-D in TBS (pH 7.4) as a function of peptide concentration.

Peptides with greater positive surface charge are known to have a higher binding affinity toward the negatively charged cancer cell membrane, and thus show enhanced anticancer efficacy.^13^ Moreover, particles with greater positive surface experience strong charge repulsions in solvents, which can promote self-stabilization among monomers as in colloidal dispersions. The zeta-potential (*ζ*) of 10 μM HAL-2, HAL-B, HAL-C and HAL-D in TBS were measured to be 3.4 ± 0.1 mV, 5.5 ± 0.2 mV, 24.5 ± 0.1 mV, and 18.7 ± 1.1 mV, respectively (Fig. 2). We note that the zeta-potential of HAL-C (*ζ*: 24.5 ± 0.1 mV) is the highest among all the peptides. We tested the concentration-dependent zeta potential of HAL-2, HAL-B, HAL-C and HAL-D. All peptides showed very slight differences in zeta-potential that do not scale as a function of concentration (Fig. 2a). The concentration-independent zeta potential of HAL peptides, particularly HAL-C and HAL-D, suggests that the formation of self-assembled HAL-C and HAL-D structures can be achieved in biologically-relevant media such as TBS. Furthermore, the relatively high zeta-potential of HAL-C increases inter-molecular interactions between HAL-C peptides, and cholesterol subunits further promote nanoparticle formation *via* hydrophobic interactions, which help stabilize the self-assembled HAL-C nanoparticles. An MBCD titration experiment, which binds to and shields cholesterol, shows that decreasing cholesterol–cholesterol interactions affects the zeta-potential of the nanoparticle solution (Fig. S5, ESI†), which further indicates that the hydrophobic interactions between cholesterol units can help stabilize the nanoparticles.

Conformational stability of peptides in TBS were further evaluated by circular dichroism (CD) spectroscopy over a 5 to 30 μM concentration range. HAL-C and HAL-D display standard α-helical bands with two negative peaks at 208 and 222 nm, while HAL-2 remains a random coil structure as indicated by a negative CD band at 205 nm. HAL-B shows sheet-like structures. The helix propensity^14^ of HAL-2, HAL-C, HAL-D are ∼10%, 90%, and 80%, respectively (Fig. 2b and S6, ESI†). Repeating CD measurements of HAL-2 (20 μM), and comparing to CD measurements of HAL-B (20 μM), HAL-C (20 μM) and HAL-D (20 μM) in TBS with pH of 4.0, 7.4, and 8.5 further confirm conformational stability of the helices under a range of pH values, and the random coil structure of HAL-2 (Fig. S7, ESI†). These results suggest that site-specific cholesterol incorporation at a peptide hydrophobic site drives the peptide to adopt a stable α-helical secondary structure, facilitated by the increased hydrophobic microenvironment – a structure absent in the unsubstituted cholesterol-free HAL-2 peptide.

**Fig. 3:**
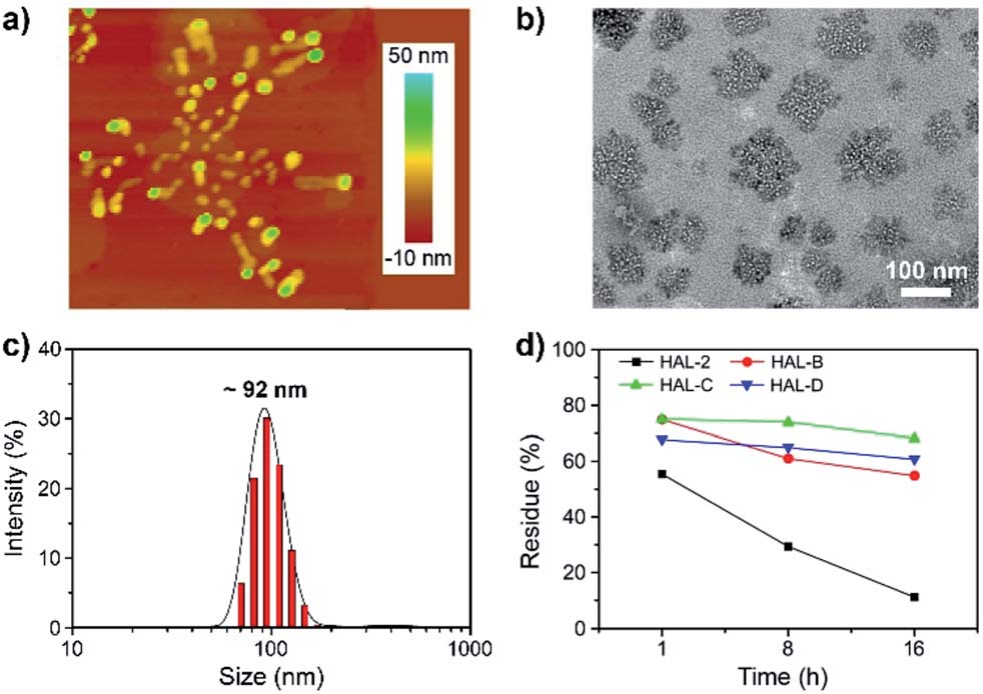
(a) AFM and (b) TEM images of HAL-C (10 μM) in TBS (pH 7.4). (c) DLS of HAL-C (10 μM) in TBS (pH 7.4). (d) HPLC spectra of peptides, serum, and peptides incubated with mouse serum taken at 1 h, 8 h and 16 h at 37 °C.

We performed dynamic light scattering (DLS) measurements of all peptides to estimate the hydrodynamic radii of the resulting self-assemblies. DLS measurements reveal that 10 μM HAL-B, HAL-C and HAL-D peptides self-assemble into nanoparticles with a mean diameter of 87 nm, 92 nm, and 97 nm, respectively, compared with HAL-2 nanoparticles that have a mean diameter of 47 nm (Fig. 3c, S8 and Table S1, ESI†) in TBS. By increasing the peptide concentration from 10 to 30 μM, the sizes of the nanoparticles formed by HAL-2 (90 nm), HAL-B (125 nm), HAL-C (121 nm) and HAL-D (104 nm) also increased. To confirm that peptides self-assemble into nanoparticles, we performed atomic force microscopy (AFM) and transmission electron microscopy (TEM) imaging to investigate the morphology and size polydispersity of the self-assembled peptides (10 μM) in TBS. Interestingly, AFM (Fig. 3a) and TEM (Fig. 3b) images show that only HAL-C (10 μM) self-assembles into stable branching nanoparticles with a sharply-distributed average diameter of ∼100 nm and a height of 10–20 nm. In contrast, we observe that HAL-2 (10 μM) forms a broad distribution of colloidal spherical particles with diameters ranging from tens of nanometers to a few microns, and heights of 50 nm in TEM and AFM images (Fig. S9a and S10a, ESI†). HAL-B and HAL-D both self-assemble into nanoparticles, with nanometer-scale diameters ranging from tens to hundreds of nanometers (Fig. S9b–d and S10b–d, ESI†), and heights of 10–20 nm. Thus, it is likely that HAL-C nanostructures self-assemble *via* inter-cholesterol subunit interactions of individual HAL-C peptides, which is absent in the original HAL-2 sequence. We stress that the α-helix folding of HAL-C is driven by the asymmetry (hydrophobic and hydrophilic) of the peptide's two faces. This folding pattern will drive a random coil structure to adopt a helical structure (Fig. 1b).^15^ AFM and TEM characterization of HAL-C shows these peptides subsequently self-assemble to form stable branched nanoparticles with a narrow diameter distribution. MD simulations show that the self-assemblies formed by HAL-2 are not stable, whereas both HAL-B and HAL-D can self-assemble into one stable nanoparticle, while HAL-C forms two small nanoparticles (Fig. S11, ESI†). MD simulations also reveal that the lysine residues lie on the surface of the nanoparticle self-assemblies of HAL-B, HAL-C and HAL-D, which promote charge repulsions between the nanoparticles. We observe some hydrophobic residues lying on the surface of self-assemblies, which can drive the self-assemblies to further aggregate. HAL-B showed the highest hydrophobic residue exposure (*ca.* 44%, Fig. S12, ESI†), HAL-C and HAL-D self-assemblies have slightly lower hydrophobic residue surface exposures (*ca.* 35% and *ca.* 33.5%, respectively, Fig. S12, ESI†). Based on the highly positive zeta potential of HAL-C and HAL-D nanoparticles, and the hydrophobic residue exposure revealed by MD simulations, it is likely that charge repulsions and hydrophobic interactions drive the final morphology of the self-assemblies. Conversely, the relatively small zeta potential of HAL-B and HAL-D promotes their self-assembly into spherical nanoparticles. HAL-C forms stable branched nanoparticles with a tightly-distributed distribution of nanoparticle diameters, likely due to the relatively strong charge repulsions between the nanoparticles. The stability of the peptides was examined by a serum test and subsequent HPLC analysis. Results show that HAL-C is the most stable peptide, with 68.3% remaining intact after incubation with serum for 16 h (Fig. 3d, S13 and Table S2, ESI†). HAL-B and HAL-D showed 54.8% and 60.7% intactness, respectively. HAL-2 is the least stable peptide, with only 11.2% of the peptide remaining intact after 16 h of incubation in serum.

**Fig. 4:**
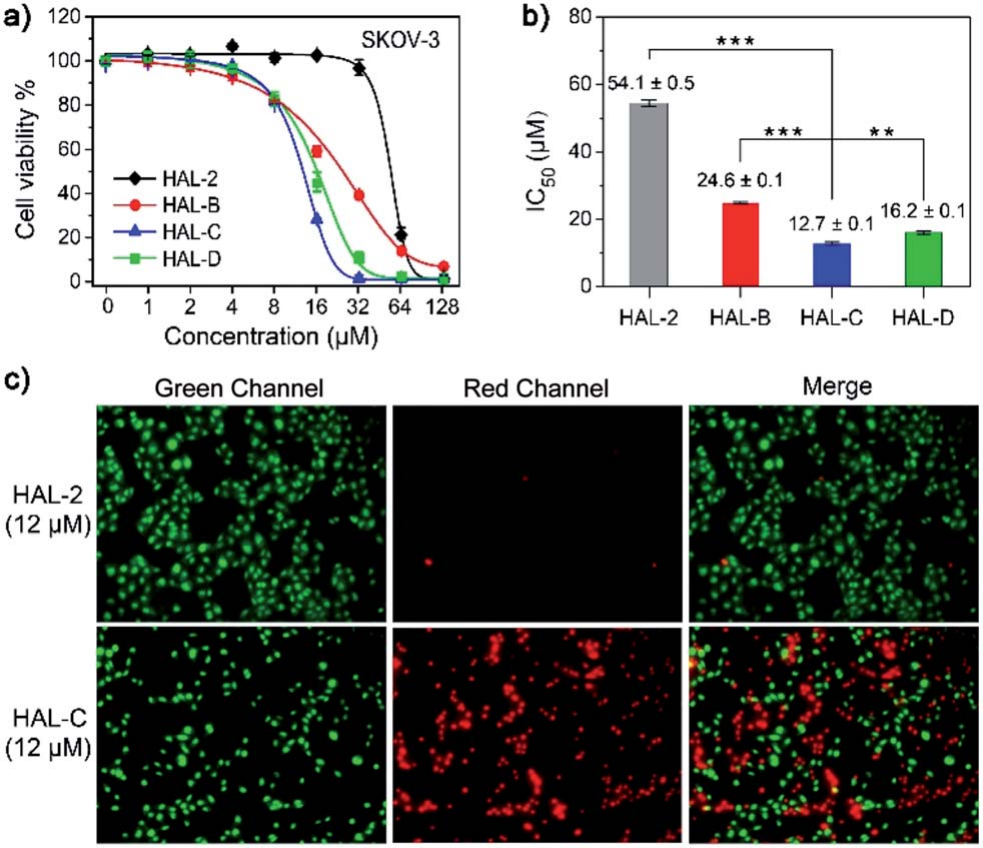
(a) Dose-response curves of HAL-2, HAL-B, HAL-C and HAL-D for SKOV-3 cells. The data are presented as mean ± SD (*n* = 5). (b) IC_50_ values of SKOV-3 cells incubated with the four peptide nanoparticles. (**P < 0.01, ***P < 0.001). (c) Live/dead assays of SKOV-3 cells incubated with HAL-2 (12 μM) and HAL-C (12 μM) imaged with CLSM.

The anticancer activity of all the peptides was tested by an MTT assay. As shown in Fig. 4a and b, for SKOV-3 ovarian cancer cells, cholesterol-modified peptides HAL-B (IC_50_ = 24.6 ± 0.1 μM), HAL-C (IC_50_ = 12.7 ± 0.1 μM) and HAL-D (IC_50_ = 16.2 ± 0.1 μM) have low IC_50_ values compared to that of unmodified HAL-2 (IC_50_ = 54.1 ± 0.5 μM). For A549 adenocarcinomic human alveolar basal epithelial cells, IC_50_ values also show that modified peptides exhibit significantly enhanced cytotoxicity towards cancer cells as compared to the native HAL-2 peptide (Fig. S14, ESI†), providing high therapeutic efficacy toward cholesterol-rich and negatively charged A549 cells.^16^ Due to the superior stability and cellular activity of the HAL-C peptide nanoparticles, we chose HAL-C for further experiments, and HAL-2 as a control. Live/dead cell staining assays of HAL-2 and HAL-C dosed SKOV-3 cells also shows that HAL-C is more potent than HAL-2.^17^ Fig. 4c shows the results of live/dead cell assays with SYTO-9/PI, in which live cells fluorescence green, and dead cells fluorescence red. We find that HAL-C induces death of SKOV-3 cells, shown by a 50% green and 50% red fluorescence cell count. In contrast, an equal concentration of HAL-2 does not induce SKOV-3 cell death, shown by a 97% green and 3% red fluorescence cell count.

**Fig. 5:**
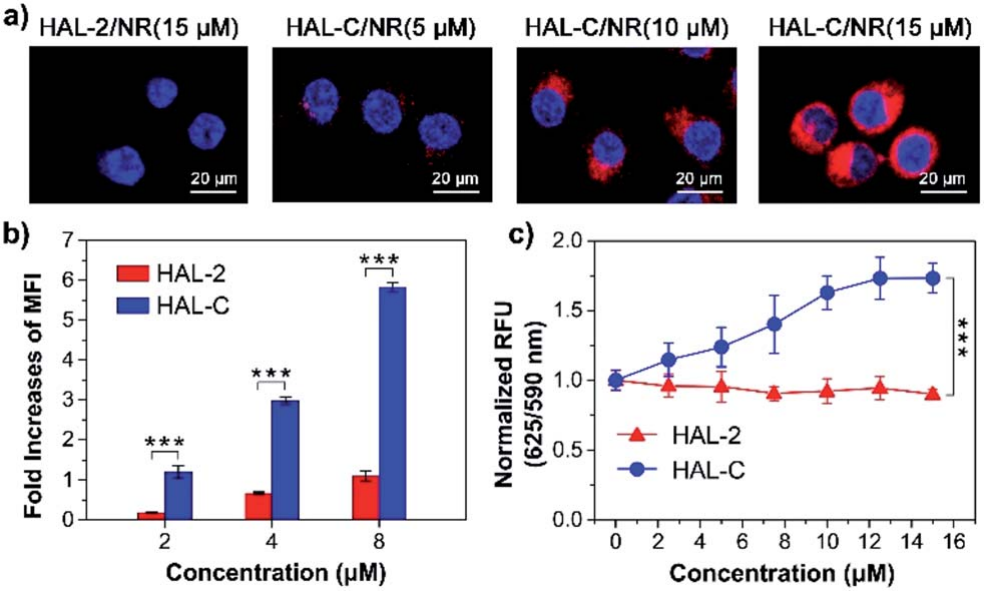
(a) CLSM images of SKOV-3 cells cultured for 1 h with the HAL-2/NR complex (15/0.2 μM), and HAL-C/NR complex at various concentrations (5/0.06, 10/0.12 and 15/0.2 μM), (NR, red), (DAPI, blue). (b) The fold-increase values of MFI for SKOV-3 cells treated with different concentrations of HAL-2/NR and HAL-C/NR complex (2/0.025, 4/0.05 and 8/0.1 μM) in 5A media, respectively. (c) Dose-response curves of HAL-2 and HAL-C in the presence of 100 μL DiBAC4 (5) for the membrane potential effects in SKOV-3 cells at various concentrations (0–15 μM). Data are normalized to DiBAC4 (5) fluorescence responses and presented as mean ± SD (*n* ≥ 3, ***P < 0.001).

Furthermore, to examine the stability of the self-assembled HAL-C nanoparticles, the critical nanoparticle forming concentration (CNC) was probed with Nile Red (NR) dye staining.^18^ The CNC of HAL-C was found to be 7 μM in TBS, suggesting that at and above 7 μM, HAL-C can self-assemble and encapsulate NR dye. Under the same conditions, HAL-2 does not reach a discernible CNC, suggesting it does not form nanoparticles and can therefore not encapsulate NR dye (Fig. S15, ESI†). The stable nanoparticles formed by HAL-C encapsulating Nile Red (NR) dye were then used to study the binding and penetration of NR-loaded HAL-C nanoparticles into SKOV-3 cells. SKOV-3 cells were incubated with HAL-2/NR (15/0.2 μM) and HAL-C/NR (5/0.06, 10/0.12 and 15/0.2 μM) for 1 h at 37 °C in McCoy's 5A media. Co-localization fluorescence imaging was used to visualize NR dye relative to the cell, and DAPI staining allowed localization of NR dye relative to the cell nucleus. Cell images were obtained by confocal laser scanning microscopy (CLSM) (Fig. 5a). SKOV-3 cells treated with HAL-C/NR exhibit strong red fluorescence, with the NR fluorescence intensity increasing with increasing HAL-C/NR concentration. In contrast, we find no NR dye fluorescence in SKOV-3 cells treated with HAL-2/NR. Flow cytometric assays (Fig. 5b and S16, ESI†) also revealed a concentration-dependence of NR fluorescent signals when added with, and thus encapsulated by HAL-C, but not when the same dye was incubated with HAL-2. To further investigate the mechanism of cell penetration, cell membrane potentials were tested by monitoring the fluorescence intensity of the anionic cell membrane marker DiBAC4 (5).^19^ As shown in Fig. 5c, SKOV-3 cells incubated with 0 to 16 μM HAL-2 show no change in membrane potential. However, for SKOV-3 cells incubated with 0 to 15 μM HAL-C, the fluorescence intensity of the membrane increases with increasing HAL-C concentration. Our results suggest HAL-C induces depolarization of the SKOV-3 cell membrane.

**Fig. 6:**
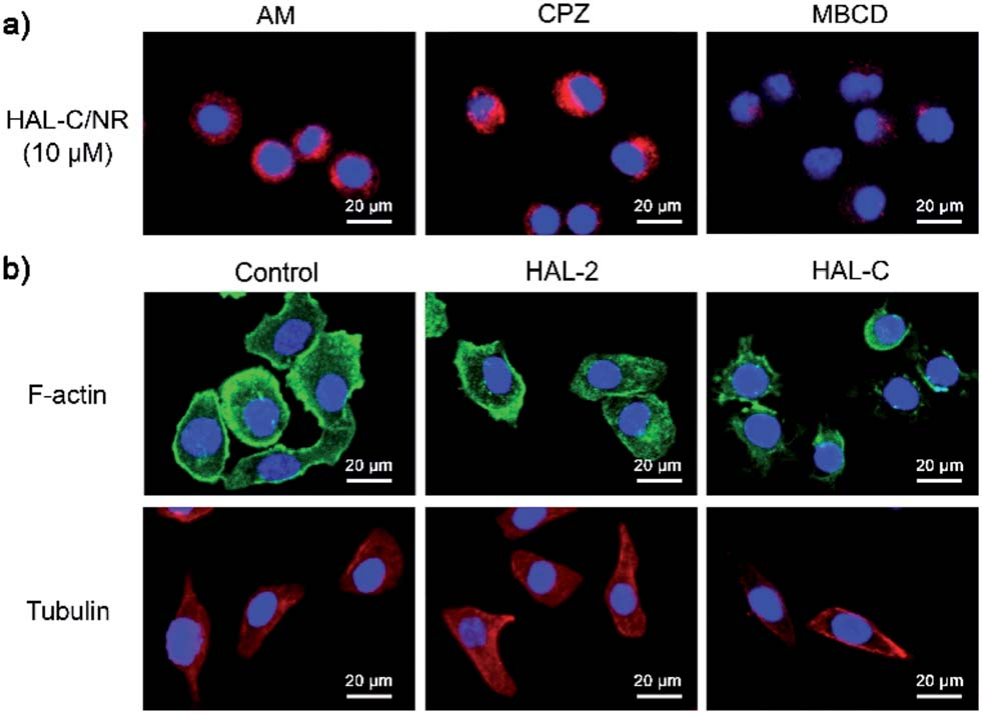
(a) CLSM images of SKOV-3 cell pre-treated with AM, CPZ or MBCD at 37 °C for 40 min, rinsed, and subsequently incubated with HAL-C/NR (10 μM) at 37 °C for 1 h. (b) SKOV-3 cells stained with Phalloidin-iFluor™ 488 (F-actin, green), DAPI (nuclei, blue), or tubulin tracker (red) after incubation with HAL-2 (10 μM) and HAL-C (10 μM) for 1 h.

To probe the pathway of cellular uptake and death by HAL-C, we quantified the delivery efficiency of HAL-C in SKOV-3 cells with various endocytosis inhibitors, including amiloride (AM, macropinocytosis inhibitor), chlorpromazine (CPZ, clathrin-mediated endocytosis inhibitor) and methyl-beta cyclodextrin (MBCD, a lipid raft-mediated endocytosis inhibitor), with concentrations of inhibitors previously reported in the literature.^20^ We observe that the intracellular delivery efficiency of HAL-C was significantly decreased only by MBCD treatment (Fig. 6a), suggesting that a lipid raft-mediated endocytosis pathway is the primary intracellular delivery mechanism of HAL-C nanoparticles. Based on MTT assays, MBCD showed no significant effects toward SKOV-3 cells at the tested concentrations within the 40 min incubation period, and we found that AM and CPZ slightly decrease SKOV-3 cell viability by 20% and 38%, respectively, due to inhibition of clathrin-mediated endocytosis and micropinocytosis, which inherently decrease cellular metabolic activity (Fig. S17, ESI†). However, all cells assayed remained structurally intact.

We observe that the intracellular delivery of HAL-C is inhibited only in MBCD-treated cells (Fig. 6a), suggesting that a lipid raft-mediated endocytosis pathway is the primary intracellular delivery mechanism of HAL-C nanoparticles. The potency of HAL-C toward MBCD treated cells is lower than for untreated cells (Fig. S18, ESI†), which indicates that cholesterols on the cell membrane play an import role in cellular uptake of HAL-C, and that HAL-C most likely targets cholesterol-rich areas of the cell membrane. Lipid-raft mediated endocytosis is driven by cholesterol-dependent invagination of glycolipid rafts, thus supporting our design strategy of targeting cholesterol-rich cancer cell membranes with cholesterol-modified HAL peptides. To further examine the mechanism of HAL-induced cell death, we probed the structure and integrity of the SKOV-3 cellular cytoskeleton upon exposure to HAL-2 and HAL-C. We examined the changes of F-actin and microtubule structure upon exposure of SKOV-3 cells exposed to 10 μM of HAL-2 or HAL-C.^21^ As shown in Fig. 6b, SKOV-3 cells treated with HAL-2 have similar F-actin and microtubule network features as untreated SKOV-3 cells, but SKOV-3 cells treated with HAL-C mainly exhibit short, truncated actin filaments and condensed microtubules surrounding the plasma membrane. The HAL-C induced disruption of SKOV-3 cellular microtubule networks indicates that HAL-C nanoparticles disrupt the dynamics of F-actin and promotes the formation of apoptotic microtubule networks resulting in cell death.^22^

**Fig. 7:**
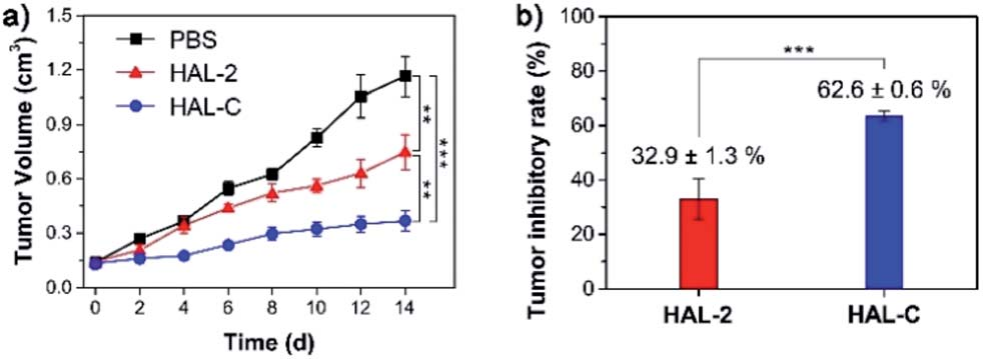
Evaluating therapeutic efficacy of HAL-C *in vivo*. (a) Time dependent relative tumor volume. (b) The tumor inhibitory rates (TIRs) of the tumors after 14 days treatment (**P < 0.01 and ***P < 0.001).

Next, the therapeutic efficacy of HAL-C was evaluated *in vivo*. Tumor mice models bearing subcutaneous SKOV-3-xenografts were dosed by intravenous injections of HAL-C (5 mg kg^–1^) and HAL-2 (5 mg kg^–1^) every 2 days. A PBS injection was used as a negative control. After two weeks of treatment, the tumor volume of mice treated with HAL-C decreases by over 66%, compared to the tumor volume of mice treated with HAL-2 (Fig. 7a and S19a, ESI†). We did not observe significant changes in mouse body mass for the mice in our study (Fig. S19b, ESI†). The tumor inhibitory rates (TIRs) were calculated from the excised mouse tumor weights. Compared with the PBS injection cohort, the TIRs of HAL-C and HAL-2 were 62.6 ± 0.6%, and 32.9 ± 1.3%, respectively (Fig. 7b). Thus, HAL-C is a promising *in vivo* tumor therapy, owing to the cholesterol modification that distinguishes the mode of activity of HAL-C from the native HAL-2 peptide, as well as the HAL-C self-assembly nanoscale size, which could leverage the enhanced permeability and retention (EPR) effect to target the tumor.^23^

## Conclusions

In summary, we report a novel strategy to tailor natural polypeptides for anticancer therapy. Herein, we accomplish tumor-cell targeting based on single amino acid cholesterol derivatives of HAL-2 peptide. We demonstrate the strong inhibitory potency of one particular cholesterol modified HAL-2 polypeptide, HAL-C, which self-assembles to form nanoparticles. HAL-C peptide nanoparticles, but not the HAL-2 peptide counterpart, show strong potency to induce cell death in SKOV-3 cells. Our results suggest that natural polypeptides can be systematically point-modified with cholesterol to identify effective anti-tumor drug candidates. The cholesterol inserted into the HAL-2 base peptide sequence significantly increases the α-helicity of the peptide and also the surface positive charge of the resulting self-assembled HAL-C nanoparticles, which greatly increase their stability in serum. Accompanying molecular dynamics simulations and cellular imaging studies suggest the enhanced cellular uptake of the modified peptide can be attributed to activation of a lipid raft-mediated endocytosis pathway that disrupts the cellular microtubule network. The self-assemblies show promise of targeting a tumor in an *in vivo* mouse model. Most importantly, this work paves the way towards further study of cholesterol-modified natural polypeptides for cancer therapy.

## Experimental section

### Zeta potential measurement

The zeta potential of peptides was measured by a Nano-ZS (Zatasizer, Malvern) instrument. Samples of HAL-2, HAL-B, HAL-C and HAL-D solution (10, 20, 30 μM) were dissolved in TBS (50 mM Tris, 50 mM NaCl, pH 7.4, 25 °C). All data were corrected by subtracting the value tested for a blank scan of the buffer system. Results were presented as mean ± SD (*n* = 3).

### Circular dichroism (CD) analysis

Circular dichroism spectra of peptides were acquired under in 50 mM TBS (50 mM Tris, 50 mM NaCl, pH 7.4, 25 °C). The circular dichroism spectra of HAL-2, HAL-B, HAL-C and HAL-D were measured at 25 °C with a J-820 spectropolarimeter (Jasco, Tokyo, Japan) equipped with a rectangular quartz cell with a path length of 0.3 cm. Spectra were recorded at a scanning speed of 10 nm min^–1^ from 190 nm to 260 nm. An average of three scans were collected for HAL-2, HAL-B, HAL-C and HAL-D.

The CD spectra were then converted to mean residue ellipticity using the following equation:^24^[*θ*]_obs_ = [*θ*]_222_ × 1000/*cln*where [*θ*]_obs_ is mean residue ellipticity [deg cm^2^ dmol^–1^], [*θ*]_222_ is the observed ellipticity corrected for the buffer at a given wavelength of 222 nm [mdeg], *c* is the peptide concentration [mM], *l* is the path length [mm] and *n* is the number of amino acids.

The average helical content of the peptide was calculated according to the formula:^25^% helix = 100([*θ*]_obs_ – [*θ*]0222)/[*θ*]100222where, [*θ*]0222 = estimated ellipticity of a peptide with 0% helicity (–1000 deg cm^2^ dmol^–1^) and [*θ*]100222 = estimated ellipticity of a 100% helical peptide (–36 500 deg cm^2^ dmol^–1^).

### Atomic force microscopy (AFM)

Images were recorded by a Veeco/DI atomic force microscope. HAL-2, HAL-B, HAL-C and HAL-D were dissolved in TBS (50 mM Tris, 50 mM NaCl, pH 7.4, 25 °C). A drop of the HAL-2, HAL-B, HAL-C and HAL-D solution (10 μM) was placed on freshly cleaved mica for 30 s and allowed to dry at room temperature. The sample was then analyzed in tapping mode.

### Transmission electron microscopy (TEM)

High-resolution images of HAL-2, HAL-B, HAL-C and HAL-D were acquired using TEM. HAL-2, HAL-B, HAL-C and HAL-D were dissolved in TBS (50 mM Tris, 50 mM NaCl, pH 7.4, 25 °C). A drop of the HAL-2, HAL-B, HAL-C and HAL-D solution (10 μM) was placed on a carbon-coated grid with holes, stained with sodium phosphotungstate (2.0 wt% aqueous solutions), and dried at room temperature. TEM characterization was performed using a JEM-2100 electron microscope (JEOL, Japan).

### Dynamic light scattering (DLS)

The DLS experiments were determined by Nano-ZS (zatasizer, Malvern) instrument, and starting solutions were filtered prior to use. Samples of HAL-2, HAL-B, HAL-C and HAL-D solution (10 and 30 μM) in TBS (50 mM Tris, 50 mM NaCl, pH 7.4, and 25 °C) in a total sample volume of 1.0 mL, respectively.

### MTT assays

The cytotoxicity was tested using an MTT assay. SKOV-3 and A549 cells growing in log phase were seeded into 96-well cell-culture plates at a cell density of 5 × 10^4^ cells per well. The cells were incubated for 12 h at 37 °C under 5% CO_2_. Solutions of HAL-2 (100.0 μL per well), HAL-B (100.0 μL per well), HAL-C (100.0 μL per well) or HAL-D (100.0 μL per well) at concentrations of 1, 2, 4, 8, 16, 32, 64, 128 μM in McCoy's 5A medium were added to the wells of the treatment group, and 100.0 μL of McCoy's 5A medium was added as negative control group. The cells were incubated for 24 h at 37 °C under 5% CO_2_. A combined solution of 5 mg mL^–1^ MTT/PBS (10 μL per well) was added to each well of the 96-well plate assay, and the cells were incubated for an additional 4 hours. Formazan extraction was performed with DMSO and quantified colorimetrically using a Synergy H4 Hybrid Microplate reader (Biotek, USA), which was used to measure the OD 490 nm (absorbance value). The following formula was used to calculate the viability of cell growth: viability (%) = (mean of absorbance value of treatment group-blank/mean absorbance value of control-blank) × 100 (MTT was purchased from Sigma-Aldrich).

### Live/dead assay

SKOV-3 cells were seeded in a 35 mm Petri dish with a glass cover slide and allowed to adhere overnight at 37 °C under 5% CO_2_. Cells were then incubated with HAL-2 or HAL-C for 24 h. Cells were stained with the live/dead assay reagents (SYTO-9/PI) and then incubated at 37 °C for 15 min. After washing with 1 mL phosphate buffered saline (PBS), cells were observed by confocal laser scanning microscopy (CLSM; Nikon A1, Japan, 60× oil-immersion objective lens). Green channel for SYTO-9: excitation: 488 nm, emission collected: 500–550 nm; red channel for PI: excitation: 561 nm, emission collected: 570–620 nm (SYTO-9/PI were purchased from Invitrogen).

### Stability studies of peptides in the serum

A 1.5 mL mixture consisting of 30 μL mouse serum, 750 μL of peptides (1 mg mL^–1^) and 1470 μL of digestion buffer (pH 8.2, 50 mM Tris, 20 mM CaCl_2_) was incubated at 37 °C for 16 h. Peptides treated with digestion buffer served as controls. Aliquots of 400 μL were withdrawn from the mixture and quenched with 400 μL of 1% trifluoroacetic acid (TFA) solution at 1 h, 8 h and 16 h. The quenched solution was analysed by reverse-phase high performance liquid chromatography (RP-HPLC).

### Critical nanoparticle-forming concentration (CNC) with Nile Red

A Nile Red solution (250 nM) was prepared in THF. HAL-2 or HAL-C were dissolved at 1 mM in PBS (pH of 7.4), then the solutions were diluted serially to obtain peptide concentrations in the range of 1.0 to 35 μM. 2 μL of Nile Red solution was added to each sample and aged for 10 h to ensure full peptide disassembly at concentrations below the critical nanoparticle formation concentration. Fluorescence-emission spectra (excitation 550 nm) were recorded for an emission range between 560 and 700 nm. The maximum intensity and respective wavelength at maximum intensity were both represented as a function of the logarithm of the peptide concentration. At concentrations close to critical nanoparticle formation concentration, we observed a sharp increase in fluorescence intensity.

### Encapsulation of Nile Red

Briefly, a stock solution of Nile Red (100 μM) was prepared in CHCl_3_, HAL-2, or HAL-C was dissolved at 400 μM in aqueous solution. While being stirred, 50 mL of Nile Red stock solution was slowly added to the HAL-2 or HAL-C solution (1 mL). This solution was then stirred for 3 h and aged for 24 h at room temperature in a closed container to allow vesicles to form. The solution was freeze-dried and then dissolved in aqueous solution (1 mL) and filtered through 0.45 μm membrane to remove insoluble Nile Red.

### Confocal laser scanning microscopy (CLSM) images

SKOV-3 cells were seeded in a 35 mm Petri dish with a glass cover slide and allowed to adhere overnight. After washing the cells with PBS (pH 7.4), the SKOV-3 cells were incubated with HAL-2/NR or HAL-C/NR in McCoy's 5A for 40 min, respectively. Cells imaging was then carried out after washing with PBS (pH 7.4, 1 mL × 5 times). Cell fluorescence images were obtained with a confocal laser scanning microscope (Nikon A1, Japan, 60× oil-immersion objective lens). Red channel for Nile red: excitation: 561 nm, emission collected: 570–620 nm; blue channel for DAPI: excitation: 405 nm, emission collected: 425–475 nm.

### Flow cytometry

The quantitative evaluation of cellular uptake of peptide nanoparticles was performed by flow cytometry (BD FACSAria). SKOV-3 cells were seeded in 6-well plates (10^6^ cells per well) and cultured in McCoy's 5A (1 mL) for 12 h at 37 °C under 5% CO_2_. After that, HAL-2/NR or HAL-C/NR dispersed in McCoy's 5A (1 mL) with different peptide concentrations ranging from 2 μM to 8 μM added to the cells, with incubation at 37 °C for 40 min. The medium was then removed, and the cells were washed with PBS (1 mL × 5 times). Afterward, the cells were digested with trypsin and collected in centrifuge tubes by centrifugation. The supernatant was discarded and PBS was added to suspend the cells for examination by flow cytometry. Cells untreated with HAL-2/NR and HAL-C/NR were used as negative controls. The fluorescence scan was performed with 5.0 × 10^4^ cells. The fold-increase values of mean fluorescence intensity (MFI) were calculated by the following equation: the fold increases values of MFI = MFI of treatment group – MFI of control/MFI of control.

### Membrane potential

Membrane potential changes in SKOV-3 cells were measured using the membrane potential-sensitive dye, DiBAC4 (5). Stock solutions of DiBAC4 (5) were prepared in dimethylsulfoxide and diluted in buffer before use. SKOV-3 cells were plated at 5 × 10^4^ cells/100 μL in black/clear 96-well plate for experiments, after incubation for 24 h at 37 °C under 5% CO_2_. Subsequently, growth medium was removed, and PBS (100.0 μL per well) containing 1 μM DiBAC4 (5) was added to the wells and incubated for 40 min at 37 °C. HAL-2 or HAL-C solutions were then added to the plate to achieve final peptide concentrations of 0, 2.5, 5, 7.5, 10, 12.5, 15 μM for further 30 min incubation at 37 °C. The cell culture plate was then transferred to the temperature-controlled (37 °C) compartment of the Synergy H4 Hybrid Microplate reader (Biotek, USA) where DiBAC4 (5) fluorescence was measured from the 96 wells at excitation and emission wavelengths of 590 and 625 nm, respectively. (DiBAC4 (5) was purchased from AmyJet Scientific Inc).

### Tubulin staining

SKOV-3 cells were seeded in a 35 mm Petri dish with a glass cover slide and allowed to adhere overnight at 37 °C under 5% CO_2_. The culture medium was then removed and replaced with fresh McCoy's 5A medium containing HAL-2 (10 μM) or HAL-C (10 μM) for 1 h. After the medium was removed and the cells were washed with PBS (1 mL × 3 times), 1 mL of PBS containing 4 μL staining solution was added for 1 h. The cells were then washed with PBS (1 mL × 3 times) before imaging. Cell fluorescence images were obtained with a confocal laser scanning microscope. Green channel for Tubulin-Trakcer Red: excitation: 561 nm, emission collected: 570–620 nm; blue channel for DAPI: excitation: 405 nm, emission collected: 425–475 nm (Tubulin-Trakcer Red were purchased from Beyotime Biotechnology).

### Delivery mechanisms of HAL-2 and HAL-C

SKOV-3 cells were seeded in a 35 mm Petri dish with a glass cover slide and allowed to adhere overnight at 37 °C under 5% CO_2_. After washing the cells with PBS (1 mL × 1 times), the SKOV-3 cells were incubated with PBS containing methyl-beta cyclodextrin (MBCD, 10 mM), chlorpromazine (CPZ, 20 μM) or amiloride (AM, 5 mM) for 40 min at 37 °C. After washing the cells with PBS at the 40 minute time-point (1 mL × 3 times) 1 mL of fresh McCoy's 5A medium containing HAL-2 (10 μM) or HAL-C (10 μM) was added to the cells and left to incubate for 30 min. Cell imaging was then carried out after washing the cells with PBS (1 mL × 5 times). Cell fluorescence images were obtained with a confocal laser scanning microscope. Red channel for Nile Red: excitation: 561 nm, emission collected: 570–620 nm; blue channel for DAPI: excitation: 405 nm, emission collected: 425–475 nm. (Methyl-beta cyclodextrin, chlorpromazine and amiloride were purchased from J&K Scientific Ltd.).

### *In vivo* experiments

All animal experiments were performed in agreement with guidelines set by the Institutional Animal Care and Use Committee, and conform to the guidelines for the care and use of laboratory animals. SKOV-3 cells were washed with PBS (pH 7.4), and harvested using 0.25% Trypsin/EDTA (Sigma). After centrifugation, the harvested cells were then suspended in PBS (pH 7.4). Four week-old (approximately 15 g) female BALB/c nude mice (Shanghai Slac Laboratory Animal Co. Ltd., China) were implanted subcutaneously on the right flank with 2 million SKOV-3 cells in 0.1 mL PBS (pH 7.4), and tumors developed within four weeks.

*In vivo* antitumor studies: when the tumors reached a mean volume of 50 mm^3^ after inoculation with SKOV-3 cells, the mice were randomly separated into three groups (*n* = 3/group): (1) negative control (PBS); (2) HAL-2; (3) HAL-C. The mice received intravenous peptide injections at 5 mg kg^–1^ in 0.1 mL PBS every 2 days for 14 days. During therapy, the tumor volumes and body weights were measured every two days. Length and width of tumors were measured individually using a vernier caliper. Tumor volumes were calculated using the following formula: tumor volume = length × width^2^ × 0.5. The mice were sacrificed 16 days after the treatments according to institutional guidelines. Tumors were resected, weighed, fixed in formalin and then embedded in paraffin. The therapeutic efficacy of the treatment was evaluated by the tumor inhibition rate (TIR). This was calculated using the following equation: TIR = 100% × (mean tumor weight of control group – mean tumor weight of experimental group)/mean tumor weight of control group.

## Conflicts of interest

There are no conflicts to declare.
