# Supplementary material for: Cholesterol-directed nanoparticle assemblies based on single amino acid peptide mutations activate cellular uptake and decrease tumor volume
Source: Chem Sci. 2017 Sep 11;8(11):7552–9. doi: 10.1039/c7sc02616a (PMC5676250; doi:10.1039/c7sc02616a)
Supplement: Supplementary file 1 [file SC-008-C7SC02616A-s001.pdf]

Electronic Supplementary Material (ESI) for Chemical Science.
This journal is © The Royal Society of Chemistry 2017

Cholesterol-Directed Nanoparticle Assemblies Based on Single Amino Acid Peptide Mutations Activate Cellular Uptake and Decrease Tumor Volume

Shang Li[a, #], Rongfeng Zou[a, d, #], Yaoquan Tu[d], Junchen Wu[a, b]* and Markita P. Landry [b, c]*

[a]Key Laboratory for Advanced Materials & Institute of Fine Chemicals, School of Chemistry and Molecular Engineering, East China University of Science and Technology Shanghai 200237, China.

[b]Department of Chemical and Biomolecular Engineering, University of California Berkeley, 476 Stanley Hall, Berkeley, California 94720, USA.

[c]California Institute for Quantitative Biosciences (qb3), University of California-Berkeley, Berkeley, CA 94720

[d]Division of Theoretical Chemistry and Biology, School of Biotechnology, KTH Royal Institute of Technology, SE-10691 Stockholm, Sweden

[#] These authors contributed equally to this work

Content

1. General information 4
2. Synthesis of cholesterol analogue (CA) 5
3. General procedure for synthesis of HAL-2, HAL-B, HAL-C and HAL-D 7
4. MTT assay 9
5. Zeta Potential measurement 10
6. Live/Dead Assay 10
7. Circular dichroism (CD) analysis 11
8. Transmission electron microscopy (TEM) 11
9. Atomic force microscopy (AFM) 12
10. Dynamic light scattering (DLS) 12
11. Critical nanoparticle-forming concentration by Nile Red assay 12
12. Dye loading in the peptide vesicles 12
13. Confocal laser scanning microscopy (CLSM) images 13
14. Flow cytometric assay 13
15. Membrane potential 14
16. Actin staining 14
17. Tubulin staining 15
18. Delivery mechanisms of HAL-2 and HAL-C 15
19. Stability studies in serum 15

20. *In vivo* experiments 16
21. Computer modelling 17
22. Supplemental Figures 18
23. References: 28

1. General Information:

All reactions were performed under an argon atmosphere using anhydrous freshly distilled solvents. All organic solvents were dried and distilled before use. Anhydrous tetrahydrofuran (THF) and dimethyl formamide (DMF) were distilled over Na and CaH_2, and kept anhydrous with 4Å molecular sieves. Water was purified by a Millipore filtration system. N,N-dimethylpyridin-4-amine (DMAP), N,N-Diisopropylethylamine (DIEA),1-(3-Dimethylaminopropyl)-3-ethylcarbodiimide hydrochloride (EDC·HCl), N-(2-aminoethyl)- phenylmethyl ester, N-Hydroxybenzotriazole (HOBt), N, N-diisopropylcarbodiimide (DIC), and Fmoc-protected amino acids were purchased from GL Biochem (Shanghai) Ltd. Other reagents were purchased from Shanghai Boyle Chemical CO., LTD. All reagents and chemicals are AR grade and used without further purification unless otherwise noted. Analytical thin-layer chromatography (TLC) was performed on silica gel plates using UV light as visualizing agent.

All melting points were measured with a Bruker Melting-Point B-450 apparatus with an open end glass capillary tube. The melting points were not corrected. The ^{1}H and ^{13}C NMR spectra were recorded at room temperature with a Bruker AV 400 spectrometer. High-resolution mass spectra were obtained on an LCT Premier XE (electronic spray ionization, ESI) and a Waters Micro-mass ® Q-TOF (ESI) spectrometer. The mass spectra of the peptides were tested on an AB Sciex (MALDI-TOF) mass spectrometer. The analytical "High Performance Liquid Chromatography" (HPLC) was performed with the following parameters: reversed phase, RP-C_{18} HPLC column and UV detector. The mobile phase was a gradient of 10-90% methanol aqueous solution containing 0.5‰ TFA at a total flow rate of 0.8 mL/min. The UV absorption peak at 400 nm of the elution was recorded for analysis. FT-IR spectra were taken on a NICOLET 380 FT-IR, Thermo Electron Corp. The UV/Vis and fluorescence spectra were recorded with a Varian Cary 100 Conc UV-Visible Spectrometer and a Fluoromax-4 Spectrofluorometer (HORIBA Scientific), respectively. pH was measured by a Mettler Toledo FE 20K pH meter.

2. Synthesis of Fmoc-Lys(cholesterol)-OH:

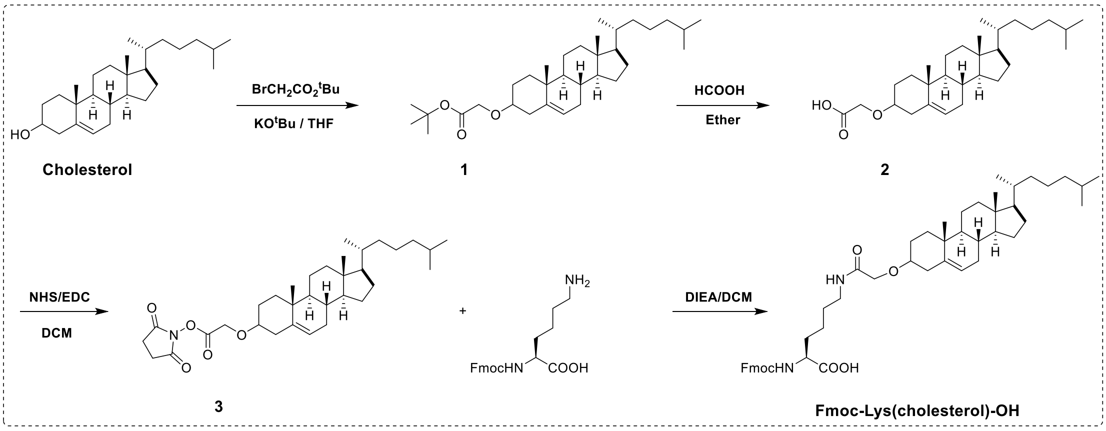

Synthesis of *t*-Butyl cholest-5-en-3β-yloxyacetate (1):

A solution of cholesterol (7.5 g, 19.4 mmol, 1.0 eq.) in THF (40 mL) was added to a mixture of potassium *t*-butoxide (8.6 g, 76.2 mmol, and 3.9 eq.) in anhydrous THF (20 mL) at 25 ºC. The mixture was stirred for 3 h. *t*-Butyl bromoacetate (5.7 mL, 38.8 mmol, 2.0 eq.) was added dropwise over 30 min, and the mixture was stirred for 14 h. Subsequently, the mixture was evaporated under reduced pressure and the residue was dissolved in DCM, washed with water and brine, dried with anhydrous Na_2SO_4. The solvent was removed under reduced pressure and the crude product was purified via flash chromatography to yield **1** (2.29 g, 24%) as a white solid. ^1H NMR (400 MHz, $CDCl_3$) δ 5.35 (m, 1H), 4.01 (s, 2H), 3.24 (m, 1H), 2.43–1.75 (m, 7H), 1.60-1.44 (m, 16H), 1.44–1.04 (m, 14H), 1.01 (s, 3H), 0.92 (d, 3H, J = 6.5 Hz), 0.87 (dd, 6H, J = 5.5 Hz), 0.68 (s, 3H). HRMS-ESI (m/z): Calcd. for $C_{33}H_{56}O_3$, 500.4229, Found $[M+Na]^+$, 523.4131

Synthesis of cholest-5-en-3β-yloxyacetic acid (2):

Formic acid (100 mL) was added to a solution of **1** (4.6 g, 9.3 mmol, 1.0 eq.) in diethyl ether (80 mL). The mixture was heated at 65 ºC for 4 h. The solvents were removed under reduced pressure to afford (3.30 g, 80%) of **2** as a white solid. ^1H NMR (400 MHz, $CDCl_3$), δ 5.37 (m, 1H), 4.13 (s, 2H), 3.33 (m, 1H), 2.43–1.44 (m, 16H), 1.44–1.04 (m, 12H), 1.01 (s, 3H), 0.92 (d, 3H, J = 6.5 Hz), 0.87 (dd, 6H, J = 5.5 Hz), 0.68 (s, 3H). HRMS-ESI (m/z): Calcd. for $C_{29}H_{48}O_3$, 444.3603, Found $[M+Na]^+$, 467.3438

Synthesis of (2, 5-Dioxopyrrolidin-1-yl) cholest-5-en-3β-yloxyacetic acid (3):

Cholest-5-en-3β-yloxyacetic acid **2** (1.0 g, 2.3 mmol, 1.0 eq.) and EDC·HCl (0.7 g, 3.4 mmol, 1.5 eq.) were dissolved in THF/DCM (1:1, 20 mL). N-hydroxysuccinimide (0.5 g, 4.5 mmol, 2.0 eq.) was added and the reaction mixture stirred at room temperature overnight. The solvent was subsequently evaporated under reduced pressure and the residue was dissolved in CH_2Cl_2, washed with water and brine, dried with anhydrous Na_2SO_4. The solvent was removed under reduced pressure, and the resulting residue was purified via flash chromatography to yield **3** (0.82 g, 67%) as a white solid. ^{1}H NMR (400 MHz, $CDCl_3$) δ 5.37 (m, 1H), 4.47 (s, 2H), 3.32 (m, 1H), 2.86 (s, 4H), 2.50-1.75 (m, 9H), 1.70-1.04 (m, 19H), 1.01 (s, 3H), 0.92 (d, 3H, *J* = 6.5 Hz), 0.87 (dd, 6H, *J* = 5.5, 1.1 Hz), 0.67 (s, 3H). HRMS-ESI (m/z): Calcd for $C_{33}H_{51}NO_5$, 541.3763, Found $[M+Na]^+$, 564. 3668

Synthesis of Fmoc-Lys(cholesterol)-OH:

Fmoc-Lys-OH (0.93 g, 1.8 mmol, 1.2 eq) and DIEA (0.6 mL, 3.0 mmol, 2.0 eq.) were dissolved in DCM (10 mL) at 0 ºC. 2, 5-Dioxopyrrolidin-1-yl- cholest-5-en-3β-yloxyacetic acid **3** (0.85 g, 1.5 mmol, 1.0 eq.) dissolved in DCM (5 mL) was added dropwise over 30 min. The solution was subsequently stirred at room temperature for 8 h. Then, the solvent was washed with water and brine, and dried with anhydrous Na_2SO_4. The solvent was removed under reduced pressure, and the resulting residue was purified via flash chromatography to yield **Fmoc-Lys(cholesterol)-OH** (0.84 g, 67%) as a white solid. Melting Piont: 71-72°C. ^{1}H NMR (400 MHz, $CDCl_3$) δ 7.73 (d, 2H), 7.60 (m, 2H), 7.38 (m, 2H), 7.29 (d, 2H), 5.79 (s, 1H), 5.30 (m, 1H), 4.42 (s, 1H), 4.34 (d, 2H), 4.18 (m, 1H), 3.99 (s, 2H), 3.50-3.10 (m, 3H), 2.50–1.75 (m, 10H), 1.70–1.04 (m, 25H), 1.01 (s, 3H), 0.92 (d, 3H, *J* = 6.5 Hz), 0.87 (dd, 6H, *J* = 5.5, 1.1 Hz), 0.67 (s, 3H). ^{13}C NMR (101 MHz, $CDCl_3$) δ 174.7, 171.4, 156.2, 143.9, 143.8, 141.3, 139.9, 127.7, 127.1, 125.3, 125.2, 122.4, 120.0, 80.3, 67.3, 67.04, 56.7, 56.1, 53.8, 53.6, 50.0, 47.2, 42.3, 42.1, 39.7, 39.5, 38.9, 38.5, 36.9, 36.7, 36.2, 35.8, 31.9, 31.8, 29.7, 29.1, 28.2, 28.0, 24.3, 23.9, 22.8, 22.6, 22.2, 21.04, 19.3, 18.7, 18.6, 17.5, 11.9, 11.8. HRMS-ESI (m/z): Calcd. for $C_{50}H_{70}N_2O_6$, 794.5234, Found $[M+Na]^+$, 817.5125.

3. Synthesis of HAL-2, HAL-B, HAL-C and HAL-D:

Fmoc Removal:

The Fmoc protecting group was treated with 20% piperidine in DMF (2×10 mL, 5 min each) under microwave radio condition (35 W, 60±5 °C, 5 min). The resin was then washed 7×10 mL with DMF (*ca.* 1 min each).

Standard Fmoc solid-phase peptide synthesis (SPPS):

Each amino acid was attached using 0.641 mmol/g loading Fmoc Rink amide resin under microwave radio (35 W, 60±5 °C, 20 min). The resin was subsequently washed with 8×10 mL DMF (*ca.*1 min each).

Cleavage from the Resin:

Cleavage of the product from the resin was achieved by treatment with a mixture of TFA/H_2O/triisopropylsilane (95:2.5:2.5) for 2 h. The cleavage mixture was collected by filtration and the resin was washed twice with pure TFA (10 mL). The filtrates were combined and concentrated under vacuum to obtain an oily residue. The peptide was precipitated by adding cold diethyl ether, followed by centrifugation of the mixture. Purity of the peptide was checked by HPLC on a RP-C_{18} column with H_2O/CH_3OH as eluent.

Fmoc-based amino acids were used as followed:

Fmoc-Gly-OH; Fmoc-Lys(Boc)-OH; Fmoc-Trp(Boc)-OH; Fmoc-Met-OH; Fmoc-Ser(tBu)-OH; Fmoc-Leu-OH; Fmoc-His(Trt)-OH; Fmoc-Ile-OH; Fmoc-Lys(cholesterol)-OH.

Synthesis of HAL-2:

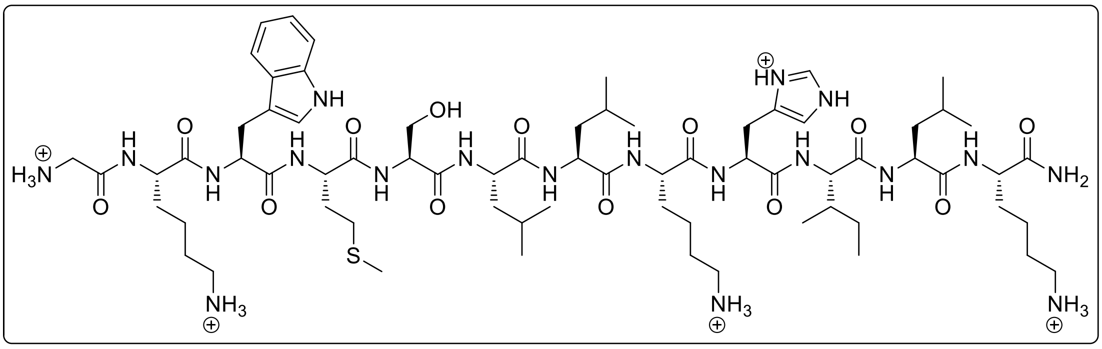

Rink amide resin (390 mg, 0.64 mmol/g, 0.25 mmol, 1.0 equiv.) was weighed out into

a plastic peptide synthesis vessel and allowed to swell in DMF (15 mL) for 2 h. The Fmoc protection group was then removed by treatment with piperidine (20%) in DMF (20 mL) under microwave radiation. After an intensive washing cycle with DMF, the following four amino acids were attached under microwave radiation for SPPS: Fmoc-Gly-OH (0.75 mmol, 3.0 equiv.), PyBOP (0.75 mmol, 3.0 equiv.) and DIMPA (1.5 mmol, 6.0 equiv.) in DMF (10 mL); Fmoc-protected amino acid (1.5 mmol, 6.0 equiv.), PyBOP (1.5 mmol, 6.0 equiv.) and DIMPA (3.0 mmol, 12.0 equiv.) in DMF (25.0 mL). The Fmoc protection group was removed by treatment with piperidine (20%) in DMF (20 mL). The product was cleaved from resin by TFA/H_2O/triisopropylsilane (95:2.5:2.5) for 3 h. After removing TFA, the peptide was precipitated by diethyl ether. The crude peptides were purified by HPLC on a RP18-column using water/CH_3CN (with 0.05% TFA) for elution. The product was identified by MALDI-TOF.

HAL-2: A white solid (30 mg, 0.02 mmol, Yield: 8.3 %, purity HPLC: 99.0%), MALDI-TOF (m/z): calcd for 1451.88, found $[M+H]^+$ 1452.70.

Synthesis of HAL-B:

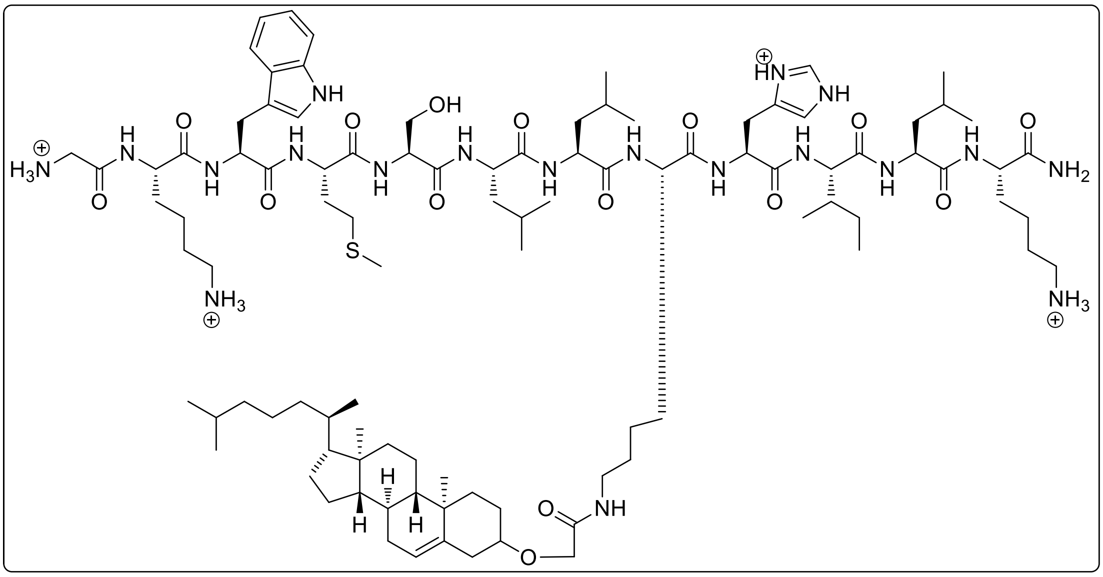

HAL-B: A white solid (17 mg, 0.008 mmol, Yield: 3.4 %, purity HPLC: 99.8%), MALDI-TOF (m/z): calcd for 1879.23, found $[M+H]^+$ 1880.14.

Synthesis of HAL-C:

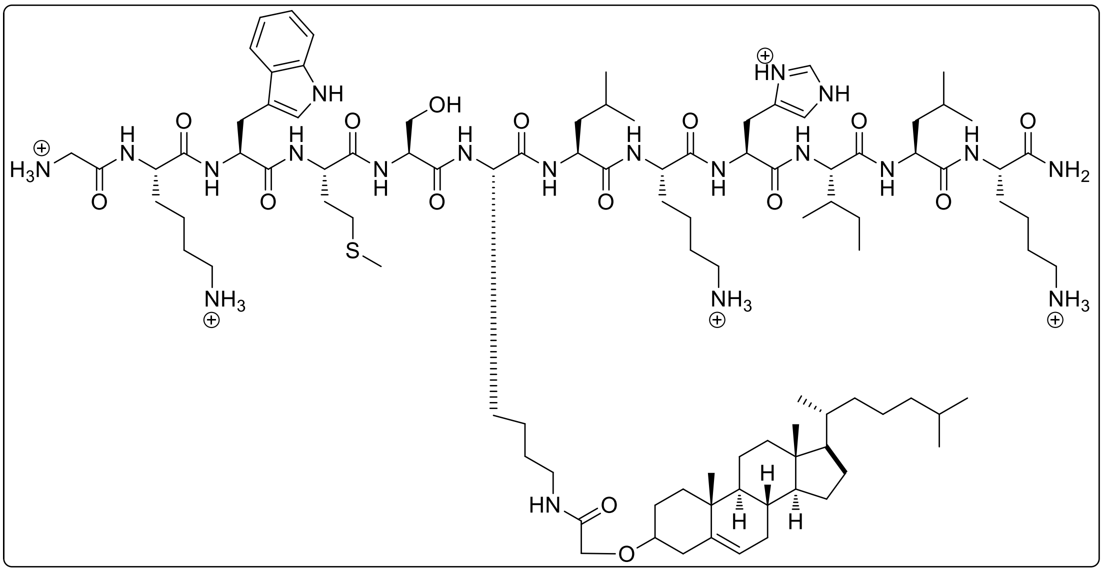

HAL-C: A white solid (34 mg, 0.018 mmol, Yield: 7.2 %, purity HPLC: 99.8%), MALDI-TOF (m/z): calcd for 1893.24, found $[M+3H]^{3+}$ 1897.27.

Synthesis of HAL-D:

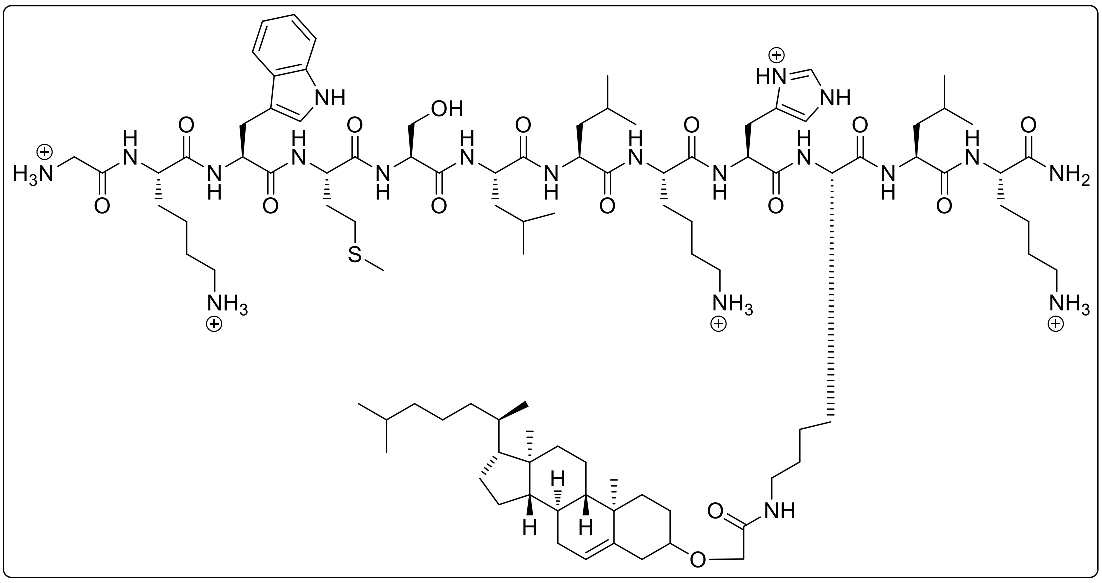

HAL-D: A white solid (10 mg, 0.005 mmol, Yield: 2.2 %, purity HPLC: 95.2%), MALDI-TOF (m/z): calcd for 1893.24, found $[M+3H]^{3+}$ 1897.40.

4. MTT assay:

Cytotoxicity was tested using an MTT assay (Sigma Aldrich). SKOV-3 and A549 Cells growing in log phase were seeded into 96-well cell-culture plates at a cell density of 5×10^4 cells/well. The cells were incubated for 12 h at 37°C under 5% CO_2. Solutions of HAL-2 (100.0 μL/well), HAL-B (100.0 μL/well), HAL-C (100.0 μL/well) or HAL-D (100.0 μL/well) at concentrations of 1, 2, 4, 8, 16, 32, 64, 128 μM in McCoy's 5A were added to the wells of the treatment group, and 100.0 μL of McCoy's 5A was added as negative control group. The cells were incubated for 24 h at 37 °C under 5% CO_2. A combined solution of 5 mg/mL MTT/PBS (10 μL/well) was added to each well of the 96-well plate assay, and the cells were incubated for an additional 4 hours. Formazan extraction was performed with DMSO and its quantity was determined colorimetrically with a Synergy H4 Hybrid Microplate reader (Biotek, USA), which was used to measure the OD_{490} nm (absorbance value). The following formula was used to calculate the viability of cell growth: Viability (%) = (mean of absorbance value of treatment group- blank /mean absorbance value of control- blank) ×100.

5. Zeta Potential measurements:

The zeta potential of peptides was measured with a Nano-ZS (Zatasizer, Malvern) instrument. Samples of HAL-2, HAL-B, HAL- C and HAL-D solution were dissolved in TBS (50 mM Tris, 50 mM NaCl, pH 7.4, 25 °C). All data were corrected by subtracting the value tested for a blank scan of the buffer system. Results were presented as mean ± SD (n = 3).

6. Live/Dead Assay:

SKOV-3 cells were seeded in a 35 mm petri dish with a glass cover slide and allowed to adhere overnight at 37 °C under 5% CO_2. Cells were then incubated with HAL-2 and HAL-C for 24 h. Cells were stained with the live/dead assay reagents (SYTO-9/PI) and then incubated at 37 °C for 15 min. After washing with 1 mL phosphate-buffered saline (PBS), cells were imaged by confocal laser scanning microscopy (CLSM; Nikon A1, Japan, 60×oil-immersion objective lens). Green channel for SYTO-9: excitation: 488

nm, emission collected: 500-550 nm; Red channel for PI: excitation: 561 nm, emission collected: 570~620 nm. (SYTO-9/PI were purchased from Invitrogen)

7. Circular dichroism (CD) analysis:

Circular dichroism spectra were acquired under inert conditions of 50 mM TBS (50 mM Tris, 50 mM NaCl, pH 7.4, 25 °C). The circular dichroism spectra of HAL-2 and HAL-C were measured at 25 °C with a J-820 spectropolarimeter (Jasco, Tokyo, Japan) equipped with a rectangular quartz cell with a path length of 0.3 cm. Spectra were recorded at a scanning speed of 10 nm/min from 190 nm to 260 nm. An average of three scans was collected for HAL-2, HAL-C and HAL-D.

The acquired CD signal spectra were then converted to mean residue ellipticity with the following equation: [1]

$$[\theta]_{obs} = [\theta]_{222} \cdot 1000/c \cdot l \cdot n$$

where $[\theta]_{obs}$ is mean residue ellipticity [deg cm^2 $dmol^{-1}$], $[\theta]_{222}$ is the observed ellipticity corrected for the buffer at a given wavelength of 222 nm [mdeg], c is the peptide concentration [mM], l is the path length [mm] and n is the number of amino acids.

The average helical content of the peptide was calculated according to the formula: [2]

$$\%helix = 100([\theta]_{obs} - [\theta]^{0}_{222})/[\theta]^{100}_{222}$$

where, $[\theta]^{0}_{222}$ = estimated ellipticity of a peptide with 0% helicity (− 1000 deg$\cdot$$cm^2$$\cdot$$dmol^{-1}$) and $[\theta]^{100}_{222}$ = estimated ellipticity of a 100% helical peptide (− 36500 deg$\cdot$$cm^2$$\cdot$$dmol^{-1}$).

8. Transmission electron microscopy (TEM):

High-resolution images of HAL-2, HAL-C and HAL-D were acquired using TEM. HAL-2 or HAL-C was dissolved in TBS (50 mM Tris, 50 mM NaCl, pH 7.4, 25 °C). A drop of the HAL-2, HAL-B, HAL-C and HAL-D solutions (10 µM) were placed on a carbon-coated grid and stained with sodium phosphotungstate (2.0 wt% aqueous solutions) and dried at room temperature. TEM characterization was performed using a JEM-2100 electron microscope (JEOL, Japan).

9. Atomic force microscopy (AFM)

Images were recorded with a Veeco/DI atomic force microscope. HAL-2, HAL-B, HAL-C and HAL-D were dissolved in TBS (50 mM Tris, 50 mM NaCl, pH 7.4, 25 °C). A drop of peptide solution (10 μM) was placed on freshly cleaved mica for 30 s and allowed to dry at room temperature. The sample was then analyzed in tapping mode.

10. Dynamic light scattering (DLS):

The DLS experiments were performed with a Nano-ZS (zetasizer, Malvern) instrument, and starting solutions were filtered prior to use. Samples of 10 and 30 μM HAL-2, HAL-B, HAL-C and HAL-D were prepared in a solution of TBS (50 mM Tris, 50 mM NaCl, pH 7.4, 25 °C) in a total sample volume of 1.0 mL.

11. Critical nanoparticle-forming concentration by Nile Red assay:

A Nile Red solution (250 nM) was prepared in THF. HAL-2 and HAL-C were dissolved at 1 mM in PBS (pH of 7.4), and the solutions were subsequently diluted serially to obtain concentrations in the range of 1.0 to 35 μM. 2 μL of Nile Red solution was added to each sample and allowed to incubate for 10 h to ensure full disassembly at concentrations below the critical aggregation concentration of the peptide. Fluorescence-emission spectra (excitation 550 nm) were recorded for an emission range between 560 and 700 nm. The maximum intensity and respective wavelength at maximum intensity were both represented as a function of the logarithm of the concentration. At concentrations close to critical aggregation concentration, we observed a sharp increase in fluorescence intensity.

12. Dye loading in the peptide vesicles[3]:

A stock solution of Nile Red (100 μM) was prepared in $CHCl_3$, HAL-2, or HAL-C was dissolved at 400 μM in aqueous solution. While being stirred, 50 mL of Nile Red stock solution was slowly added in HAL-2 or HAL-C solution (1 mL). This solution was then stirred for 3 h and allowed to incubate for 24 h at room temperature in a closed container to allow vesicles to form. The solution was freeze-dried and then dissolved in aqueous

solution (1 mL) and filtered through a 0.45 μm membrane to remove insoluble Nile Red prior to experimentation.

13. Confocal laser scanning microscopy (CLSM) images:

SKOV-3 cells were seeded in a 35 mm petri dish with a glass cover slide and allowed to adhere overnight. After washing the cells with PBS (pH 7.4), the SKOV-3 cells were incubated with HAL-2/NR or HAL-C/NR in McCoy's 5A for 40 min, respectively. Cell imaging was then carried out after washing with PBS (pH 7.4, 1 mL × 5 times). Cell fluorescence images were obtained with a confocal laser scanning microscope (Nikon A1, Japan, 60×oil-immersion objective lens). Red channel for Nile red: excitation: 561 nm, emission collected: 570-620 nm; Blue channel for DAPI: excitation: 405 nm, emission collected: 425-475 nm.

14. Flow cytometric assay

The quantitative evaluation of cellular uptake was performed by flow cytometry (BD FACSAria). SKOV-3 cells were seeded in 6-well plates (10^6 cells/well) and cultured in McCoy's 5A (1 mL) for 12 h at 37 ºC under 5% CO_2. Subsequently, HAL-2/NR or HAL-C/NR dispersed in McCoy's 5A (1 mL) with concentrations ranging from 2 μM to 8 μM was added and the cells were incubated at 37°C for 40 min. The medium was removed and the cells were washed with PBS (1 mL × 5 times). Afterward, the cells were digested by trypsin and collected in centrifuge tubes by centrifugation. The supernatant was discarded and PBS was added to suspend the cells. The suspended cells were examined by flow cytometry. Cells untreated with HAL-2/NR and HAL-C/NR were used as a negative control. The fluorescence scan was performed with 5.0×10^4 cells. The fold increase values of mean fluorescence intensity (MFI) were calculated by the following equation: The fold increases values of MFI = MFI of treatment group-MFI of control / MFI of control.

15. Membrane potential:

Membrane potential changes in SKOV-3 cells were measured using the membrane

potential-sensitive dye, DiBAC4 (5). Stock solutions of DiBAC4 (5) were prepared in dimethylsulfoxide and diluted in buffer before use. SKOV-3 Cells were plated at 5×10^4 cells/100 μL in black/clear 96-well plate for experiments, and subsequently incubated for 24 h at 37 ºC under 5% CO_2. Afterward, growth medium was removed, and PBS (100.0 μL/well) containing 1 μM DiBAC4 (5) was added to the wells and incubated for 40 min at 37 ºC. HAL-2 or HAL-C solutions were then added to the plate to achieve the final peptide concentrations of 0, 2.5, 5, 7.5, 10, 12.5, 15 μM for further 30 min incubation at 37 ºC. The cell culture plate was then transferred to the temperature-controlled (37 ºC) compartment of the Synergy H4 Hybrid Microplate reader (Biotek, USA) where DiBAC4 (5) fluorescence was measured from the 96 wells at excitation and emission wavelengths of 590 and 625 nm, respectively. (DiBAC4 (5) were purchased from AmyJet Scientific Inc).

16. Actin staining:

SKOV-3 cells were seeded in a 35 mm petri dish with a glass cover slide and allowed to adhere overnight at 37 ºC under 5% CO_2. The culture medium was removed, and fresh McCoy's 5A medium containing HAL-2 (10 μM) or HAL-C (10 μM,) was added to the cells. After 1 h, the medium was removed and the cells were washed with PBS (1 mL). After fixing by 4% paraformaldehyde for 30 minutes, we added 1 mL of 0.1% Triton X-100 in PBS buffer for 5 minutes. After washing the cells with PBS (1 mL × 3 times), 1 mL of PBS containing 1 μL of Phalloidin-iFluor™ 488 Conjugate was added to the cells for 1 h. Subsequently, the cells were washed with PBS (1 mL × 3 times) prior to imaging. Cell fluorescence images were obtained with a confocal laser scanning microscope. Green channel for Phalloidin-iFluor™ 488 Conjugate: excitation: 488 nm, emission collected: 500-550 nm; Blue channel for DAPI: excitation: 405 nm, emission collected: 425-475 nm. (Phalloidin-iFluor™ 488 Conjugate were purchased from AmyJet Scientific Inc)

17. Tubulin staining:

SKOV-3 cells were seeded in a 35 mm petri dish with a glass cover slide and allowed

to adhere overnight at 37 ºC under 5% CO_2. The cell culture medium was then removed and replaced with fresh McCoy's 5A medium containing HAL-2 (10 μM) or HAL-C (10 μM) for 1 h. After the medium was removed and the cells were washed with PBS (1 mL × 3 times), 1 mL of PBS containing 4 μL staining solution was added for 1 h. The cells were then washed with PBS (1 mL × 3 times) before imaging. Cell fluorescence images were obtained with a confocal laser scanning microscope. Green channel for Tubulin-Trakcer Red: excitation: 561 nm, emission collected: 570-620 nm; Blue channel for DAPI: excitation: 405 nm, emission collected: 425-475 nm. (Tubulin-Trakcer Red were purchased from Beyotime Biotechnology)

18. Delivery mechanisms of HAL-2 and HAL-C:

SKOV-3 cells were seeded in a 35 mm petri dish with a glass cover slide and allowed to adhere overnight at 37 ºC under 5% CO_2. After washing the cells with PBS (1 mL × 1 times), the SKOV-3 cells were incubated with PBS containing methyl-beta cyclodextrin (MBCD, 10 mM), chlorpromazine (CPZ, 20 μM) or amiloride (AM, 5 mM) for 40 min at 37 ºC. Subsequently, 400 μM HAL-2 or HAL-C were added to the petri dish to a final peptide concentration of 10 μM. After co-incubation of the inhibitor with the peptide for another 30 min, the culture medium was removed, and cells were washed with PBS (1 mL × 5 times). Cell fluorescence images were obtained with a confocal laser scanning microscope. Red channel for Nile red: excitation: 561 nm, emission collected: 570-620 nm; Blue channel for DAPI: excitation: 405 nm, emission collected: 425-475 nm. (Methyl-beta cyclodextrin, chlorpromazine and amiloride were purchased from J&K Scientific Ltd.)

19. Stability studies in serum:

A 1.5 mL mixture consisting of 30 μL serum, 750 μL of peptides (1 mg/mL), and 1470 μL of digestion buffer (pH 8.2, 50 mM Tris, 20 mM $CaCl_2$) was incubated at 37 °C for 16 h. Peptides treated with digestion buffer served as controls. Aliquots of 400 μL were withdrawn from the mixture and quenched with 400 μL of 1% trifluoroacetic acid (TFA) solution at 1 h, 8 h and 16 h. The quenched solution was analyzed by reverse-phase

high performance liquid chromatography (RP-HPLC).

20. *In vivo* experiments:

All animal experiments were performed in agreement with the guidelines of the Institutional Animal Care and Use Committee and conformed to the guide for the care and use of laboratory animals. SKOV-3 cells were washed with PBS (pH 7.4), and harvested using 0.25% Trypsin/EDTA (Sigma). After centrifugation, the harvested cells were then suspended in PBS (pH 7.4). Four-week-old (approximately 15 g) female BALB/c nude mice (Shanghai Slac Laboratory Animal Co. Ltd., China) were implanted subcutaneously on the right flank with 2 million SKOV-3 cells in 0.1 mL PBS (pH 7.4), and tumors developed within four weeks.

***In vivo* antitumor studies:** When the tumors reached a mean volume of 50 mm^3 after inoculation of SKOV-3 cells, the mice were randomly separated into three groups (N=3/group): (1) negative control (PBS); (2) HAL-2; (3) HAL-C. The mice received intravenous injections of 5 mg/kg in 0.1 mL PBS every 2 days for 14 days. During therapy, the tumor volumes and body weights were measured every two days. Length and width of tumors were measured individually using a Vernier caliper. Tumor volumes were calculated using the following formula: tumor volume = length × $width^2$ × 0.5. The mice were sacrificed 16 days after the treatments according to institutional guidelines. Tumors were resected, weighed, fixed in formalin and then embedded in paraffin. The therapeutic efficacy of the treatment was evaluated by the tumor inhibition rate (TIR). This was calculated using the following equation: TIR = 100% × (mean tumor weight of control group - mean tumor weight of experimental group)/mean tumor weight of control group.

For staining of tissue slices: Freshly dissected heart, liver, kidneys, lungs, stomach, spleen and tumor from mice of three treatment groups were fixed and embedded in paraffin. After being cut into 4-μm slices, the sections were deparaffinized and stained with Hematoxylin and Eosin solution. Finally, the sections were dehydrated and

mounted with Permount in a fume hood. The sections were scanned by a microscope (Nikon, Japan).

21. Computer modeling:

HAL-C was built with the α-helix conformation using Maestro 10.2 (Schrödinger, 2015). Then the structure was energy-minimized based on the OPLS (optimized potentials for liquid simulations) 2005 force field, choosing water as solvent. Subsequently, 10 molecules were placed with the cholesterol forming a hydrophobic core. Energy minimization was performed to the system to remove any incorrect Van der Waals contacts. This modelling is implemented for illustration.

MD simulation (system setup): Force field parameters of residue Z (with its capped structure) were obtained from the cgenff web server (https://cgenff.paramchem.org). The initial structure of the simulated systems was constructed as follows: build one peptide structure lineally, then the peptide was subjected to a short production run (10 ns) with explicit water under 500K to generate 100 random structures. After that, 10 structures were chosen randomly and put into a simulation box. The system consists of 10 peptides solvated with TIP3P water molecules, and 0.15 M NaCl was added to mimic a biological environment. A 10000-step energy minimization was applied to the system to remove any unfavorable contacts, followed by 100 ps NVT and 200 ps NPT equilibrium. The production run lasted for 250 ns. Each system was simulated twice.

Calculation of hydrophobic exposure rate: The last 50 ns of the simulation was analyzed to calculate the hydrophobic exposure rate.

The hydrophobic exposure rate was defined by the following formula:

$H = (SASA_{hydrophobic} - (SASA_{hydrophobic} + SASA_{other} - SASA_{total}) / 2) / SASA_{total} \times 100\%$;

Here $SASA_{hydrophobic}$ is the SASA of hydrophobic residues, $SASA_{other}$ is the SASA of residues other than the hydrophobic ones, $SASA_{total}$ is the SASA of all the residues.

22. Supplemental Figures:

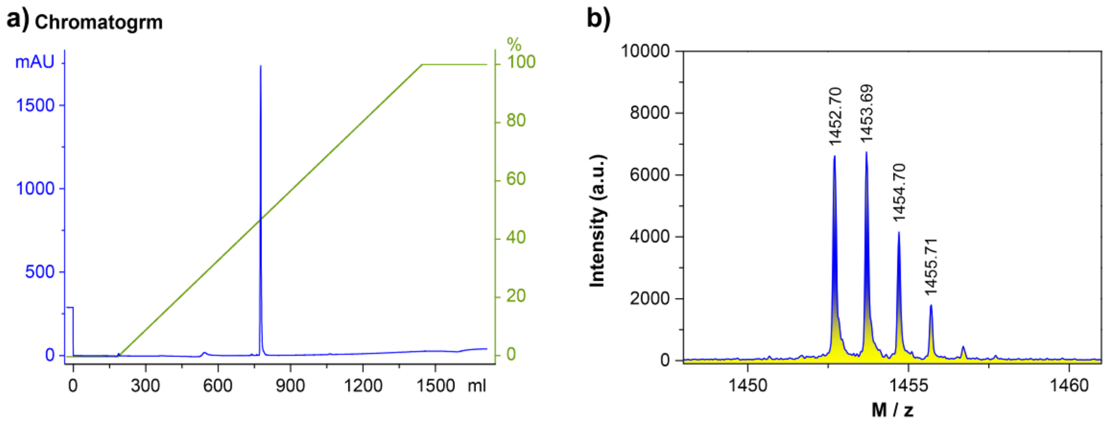

Figure S1. a) HPLC and b) MALDI-TOF MS analysis of HAL-2

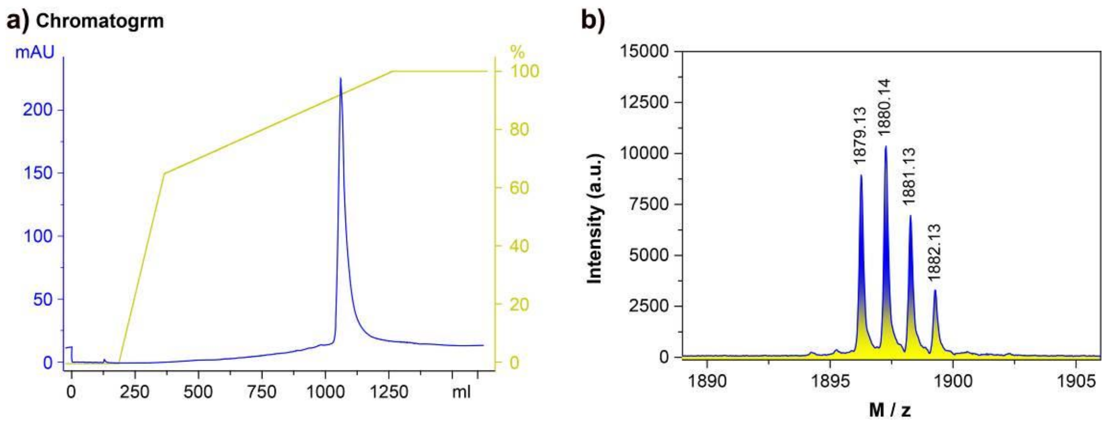

Figure S2. a) HPLC and b) MALDI-TOF MS analysis of HAL-B.

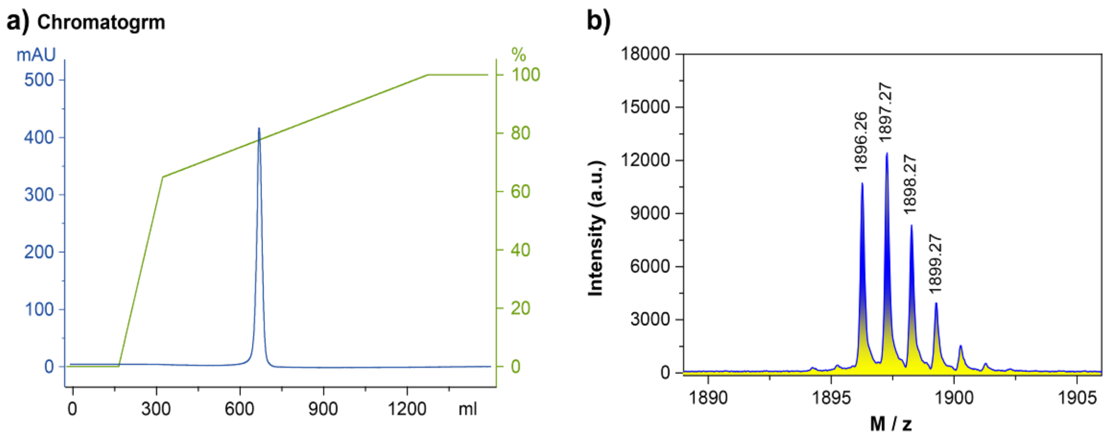

Figure S3. a) HPLC and b) MALDI-TOF MS analysis of HAL-C.

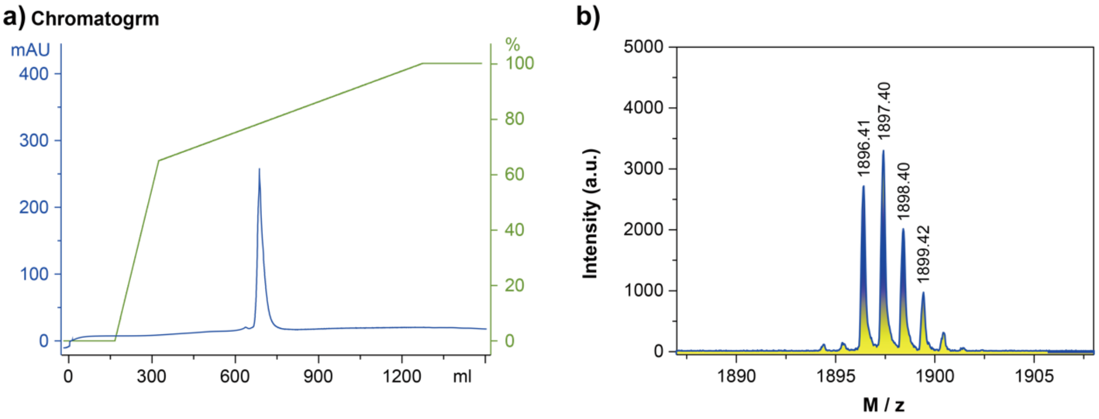

Figure S4. a) HPLC and b) MALDI-TOF MS analysis of HAL-D.

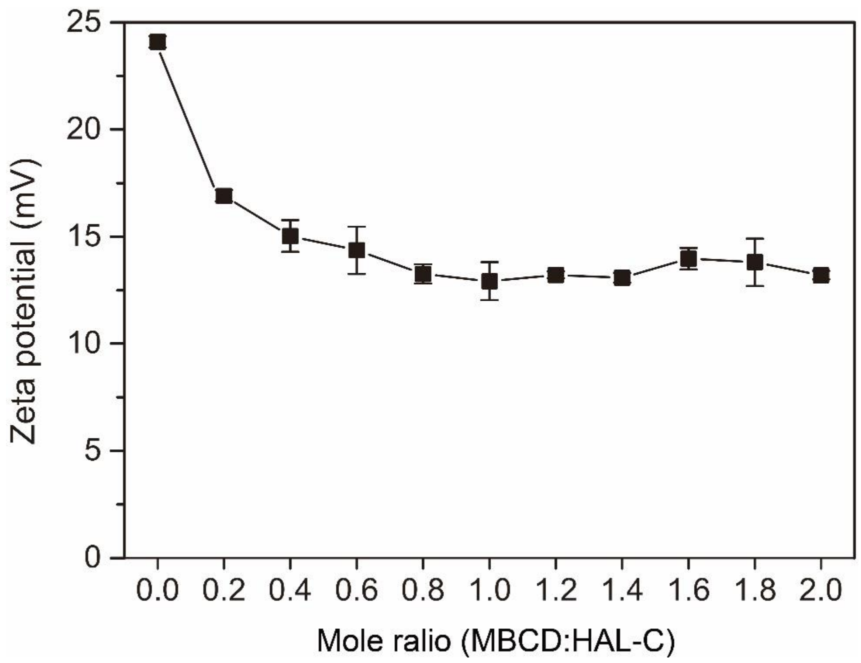

Figure S5. Zeta potentials of MBCD and HAL-C at different mole ratio in TBS (pH 7.4).

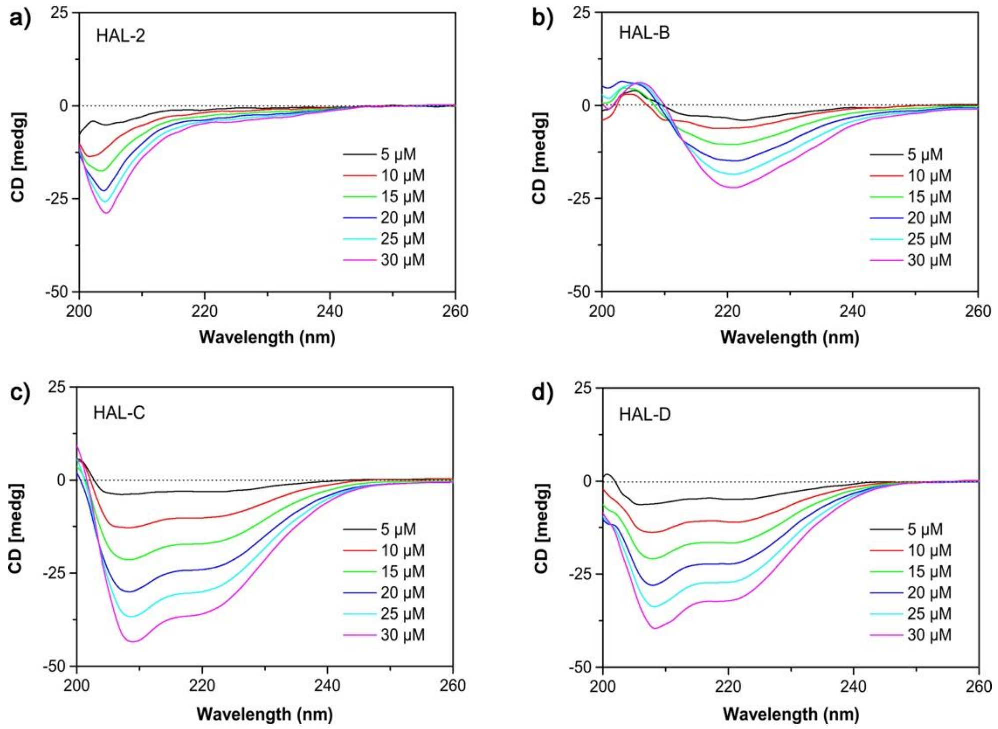

Figure S6. The CD spectra of the peptides. a) HAL-2, b) HAL-B, c) HAL-C and d) HAL-D were dissolved in TBS (50 mM Tris, 50 mM NaCl, pH 7.4, 25 °C) at different concentrations (from 5 to 30 μM).

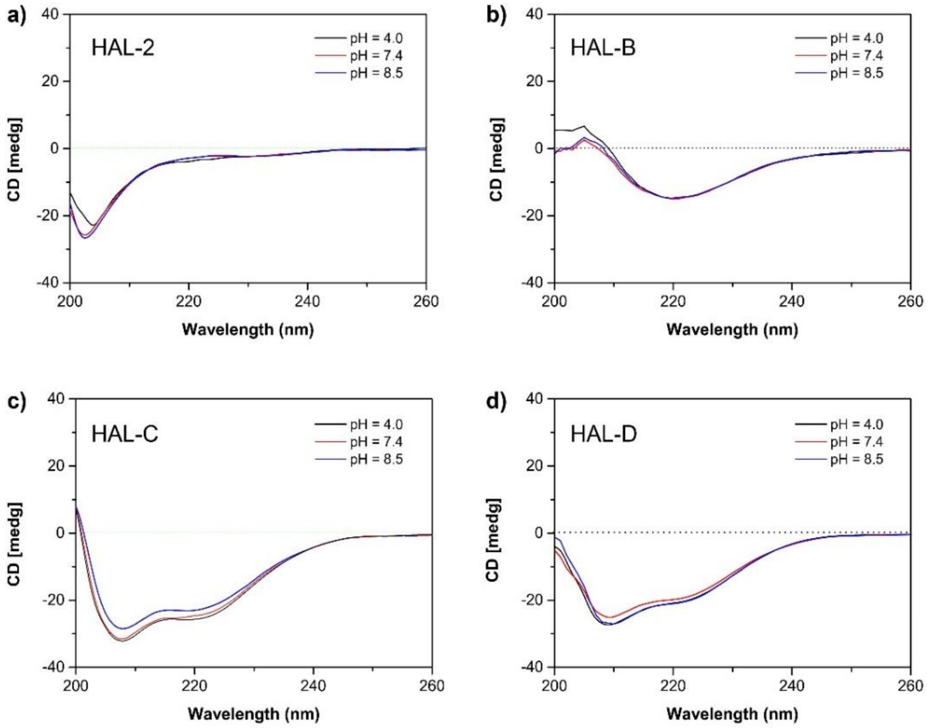

Figure S7. The CD spectra of the peptides. a) HAL-2 (20 μM), b) HAL-B (20 μM), c) HAL-C (20 μM) and HAL-D (20 μM) were dissolved in TBS at different pH values (4, 7.4, 8.5)

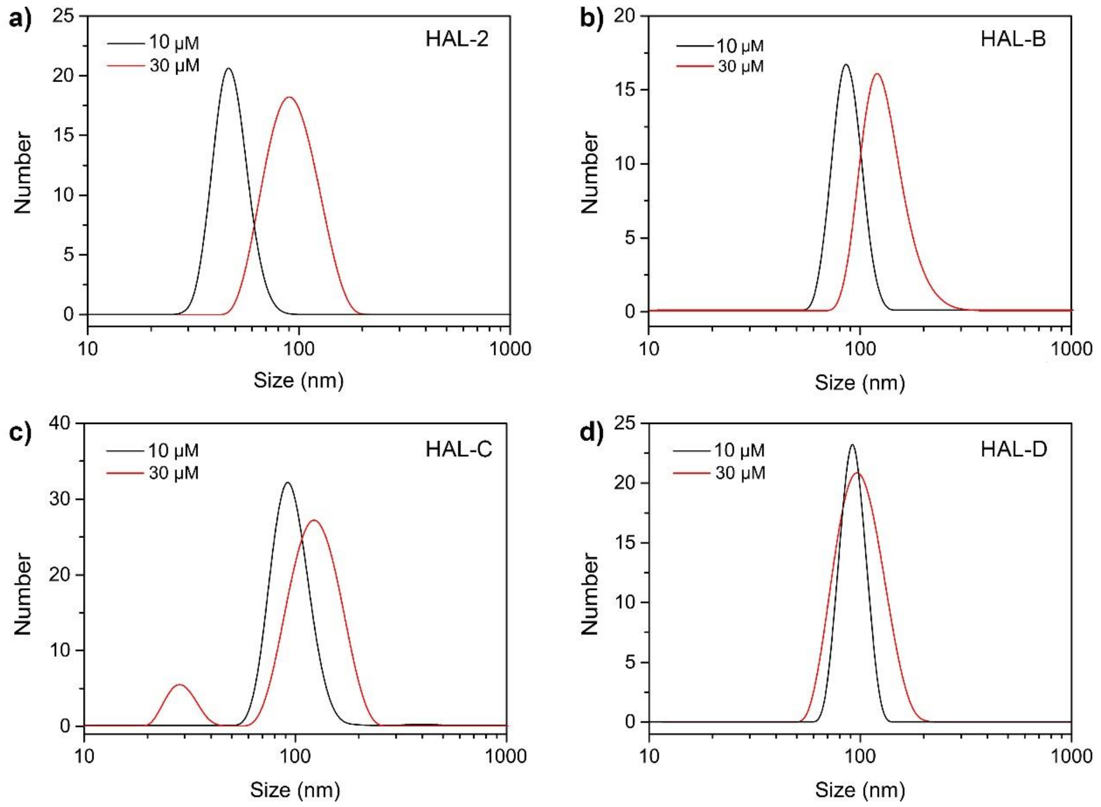

Figure S8. Dynamic Light Scattering (DLS) profile showing hydrodynamic diameters [*d* (nm)] for a) HAL-2, b) HAL-B, c) HAL-C and d) HAL-D (10, 30 µM) in TBS (50 mM Tris, pH 7.4, 25°C).

Table S1. The size of peptide self-assemblies in the TBS

Concentration	Size (nm)			
	HAL-2	HAL-B	HAL-C	HAL-D
10 µM	47	87	92	97
30 µM	90	125	121	104

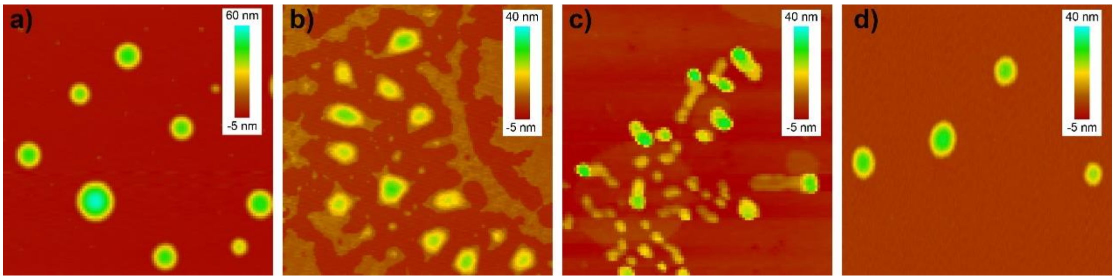

Figure S9. AFM image of a) HAL-2 (10 µM), b) HAL-B (10 µM), c) HAL-C (10 µM) and d) HAL-D (10 µM) in TBS (pH 7.4).

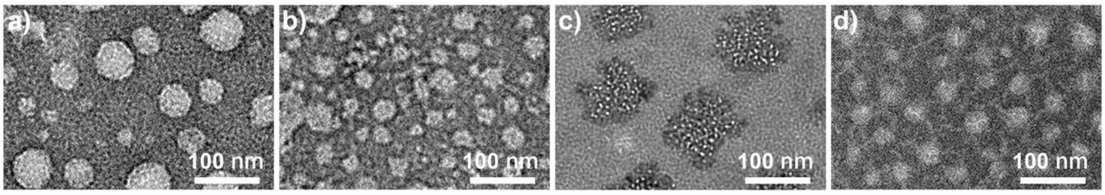

Figure S10. Transmission electron microscopy (TEM) of a) HAL-2 (10 μM), b) HAL-B (10 μM), c) HAL-C (10 μM) and d) HAL-D (10 μM) in TBS (50-mM Tris, pH 7.4, 25°C).

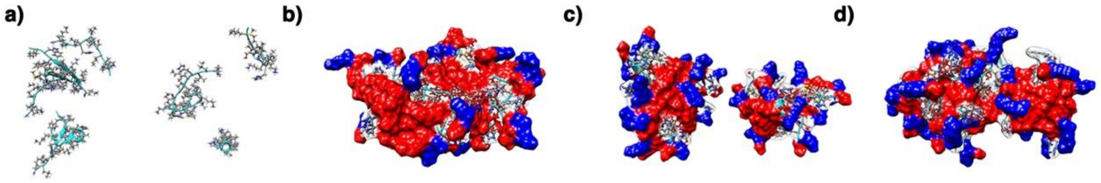

Figure S11. The final snapshots of a) HAL-2, b) HAL-B and c) HAL-C and d) HAL-D showing self-assemblies of these three peptides. Blue represents the lysine residues and red represents hydrophobic residues.

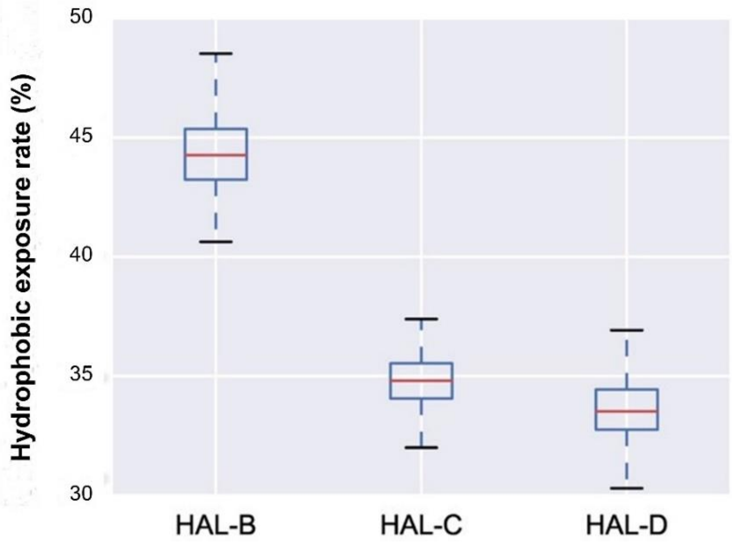

Figure S12. Box plot of hydrophobic exposure rate of HAL-B, HAL-C and HAL-D. Results were obtained from MD simulations.

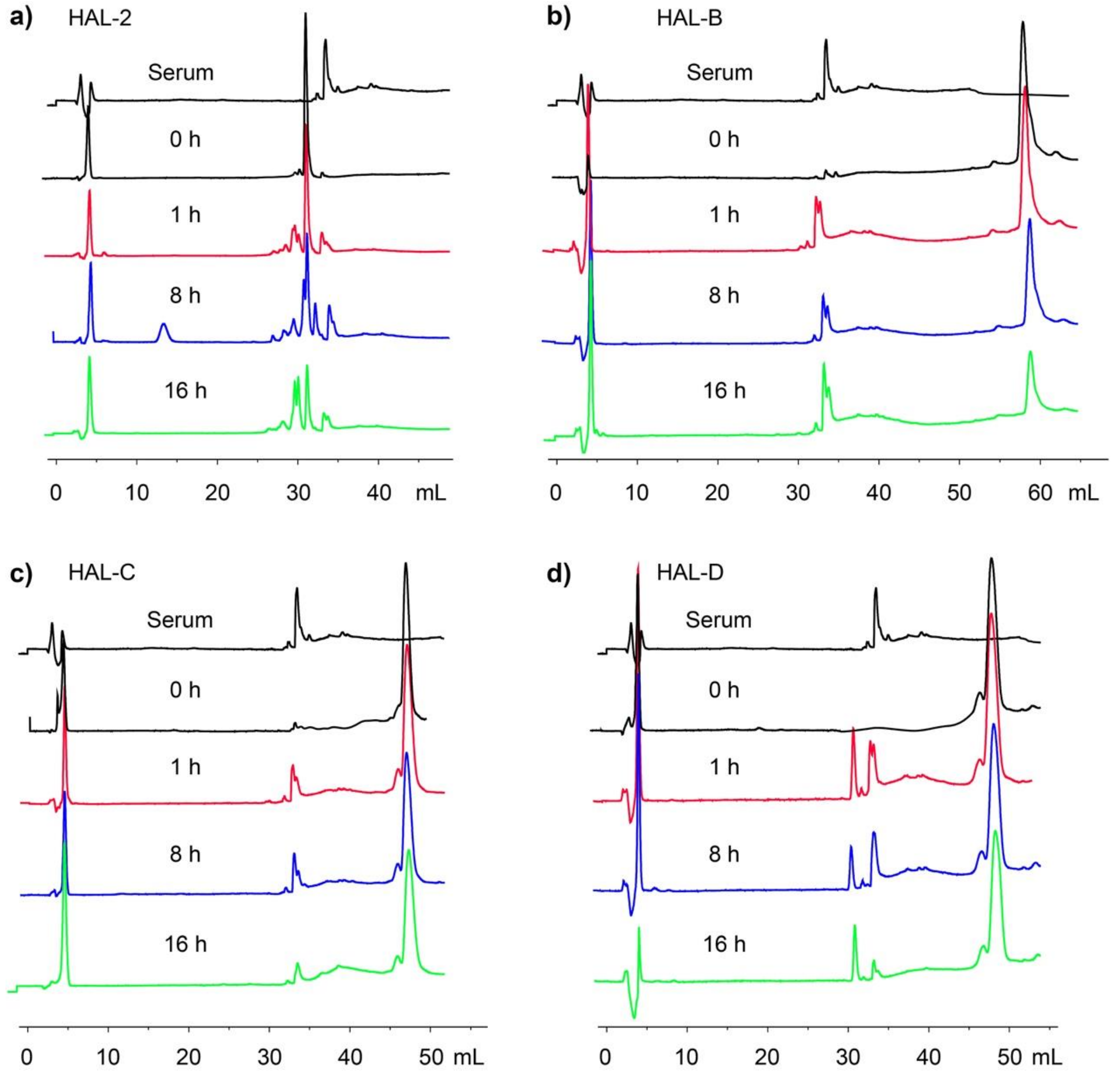

Figure S13. Analytical HPLC spectra of peptides, serum, and peptides incubated with serum taken at 1 h, 8 h and 16 h at 37°C.

Table S2. The stability of peptides in the serum

Time	Residue (%)			
	HAL-2	HAL-B	HAL-C	HAL-D
1 h	55.4	74.9	75.1	67.7
8 h	29.4	61.0	74.0	64.9
16 h	11.2	54.8	68.3	60.7

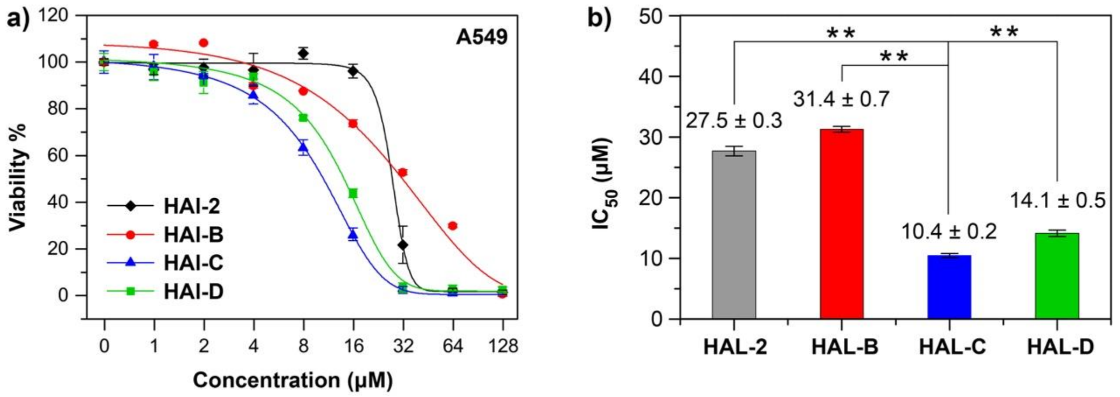

Figure S14. a) Dose-response curves of HAL-2, HAL-B, HAL-C and HAL-D for A549 cells. The data are presented as mean ± SD (n = 5). b) Shows the IC_{50} values of A549 cells. (**$P < 0.01$).

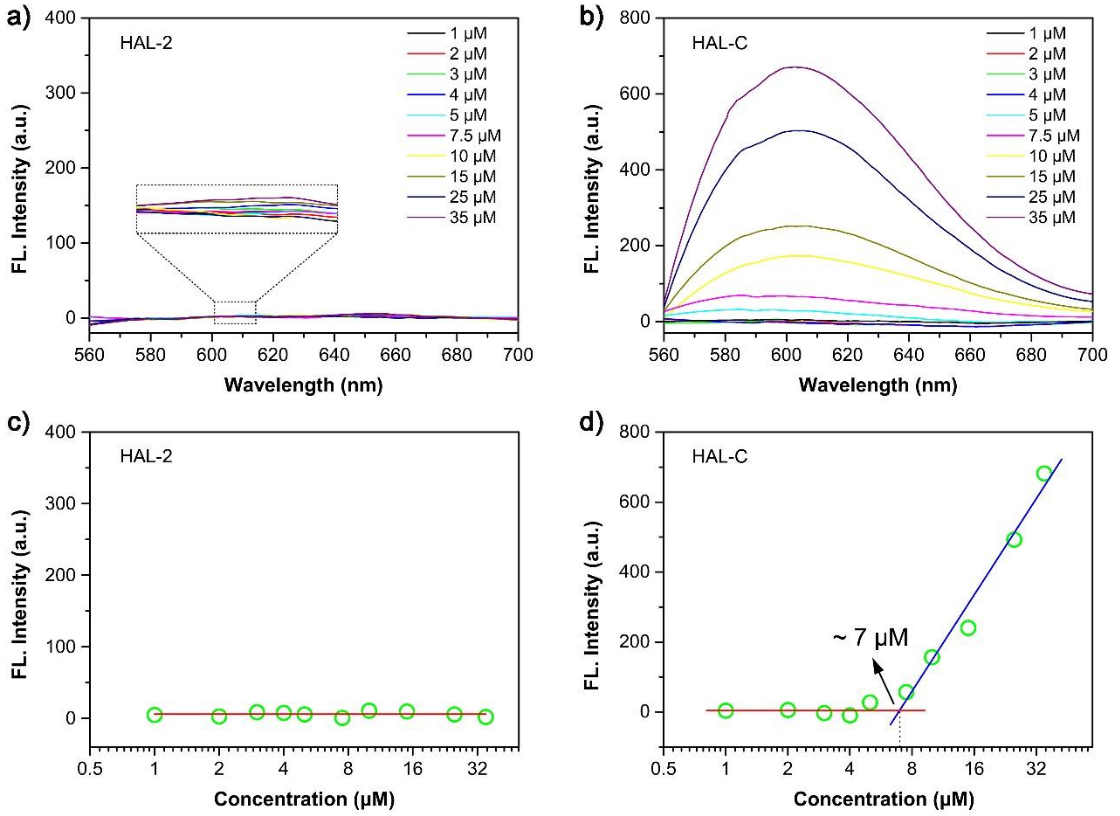

Figure S15. (a and b) Fluorescence spectra of Nile Red (250 nM) in the presence of HAL-2 and HAL-C at different concentrations (1 to 35 μM). (c and d) Dependence of fluorescence intensity of Nile Red at 600 nm on the concentration of HAL-2 and HAL-C.

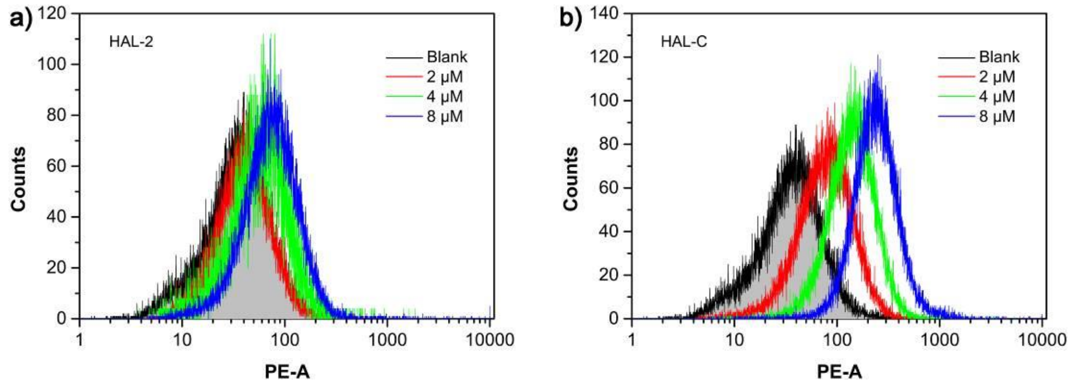

Figure S16. SKOV-3 cells treated with a) HAL-2/NR (2, 4, 8 µM) or b) HAL-C/NR (2, 4, 8 µM) at 37°C in McCoy's 5A medium, then intracellular fluorescence was analyzed by flow cytometry.

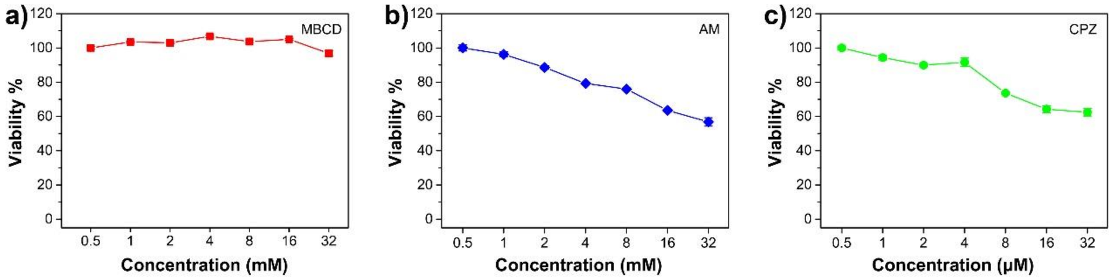

Figure S17. Dose-response curves of a) MBCD, b) AM and c) CPZ for SKOV-3 cells incubated for 40 min. The data are presented as mean ± SD (n = 5).

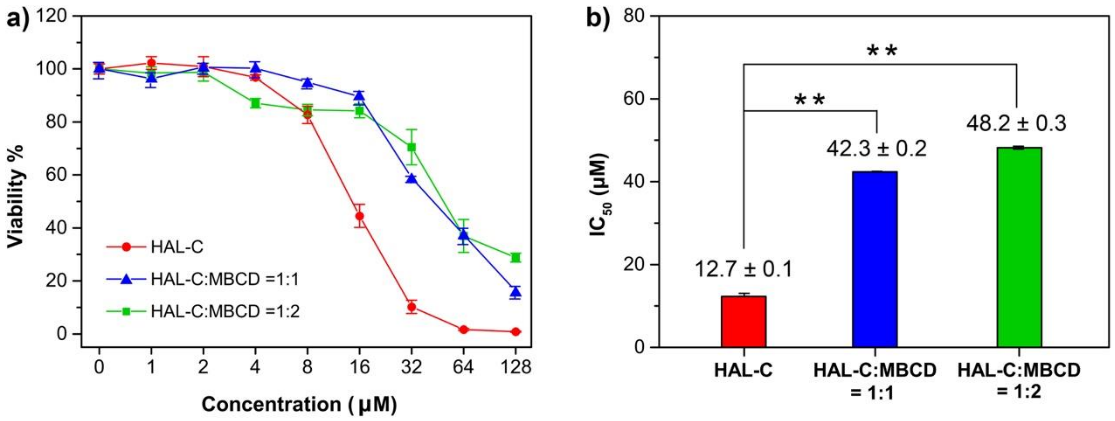

Figure S18. a) Dose-response curves of HAL-C, HAL-C/MBCD (mole ratio =1:1 and 1:2) for SKOV-3 cells. The data are presented as mean ± SD (n = 5). b) Shows the IC_{50} values of SKOV-3 cells. (**P < 0.01).

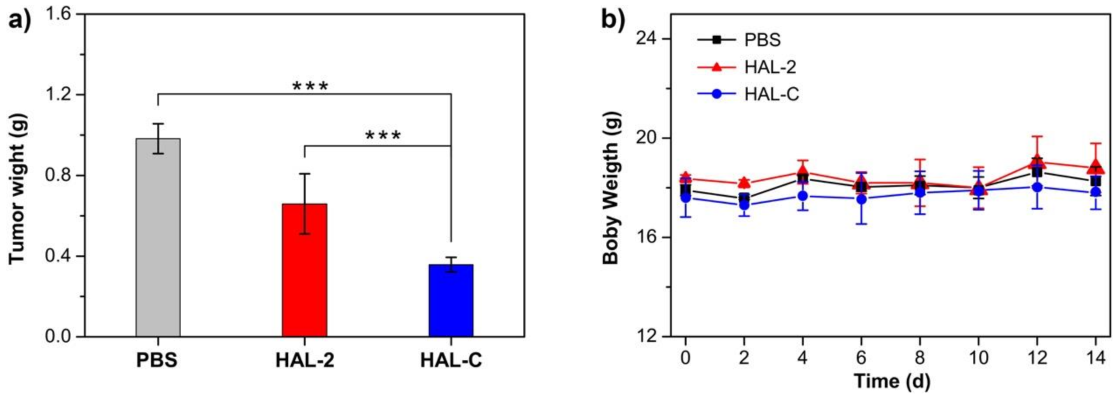

Figure S19. Evaluating therapeutic efficacy of HAL-C *in vivo*. (a) The average tumor weight after 14 days treatment. (b) Relative body weight after treatment. (***$P < 0.001$).

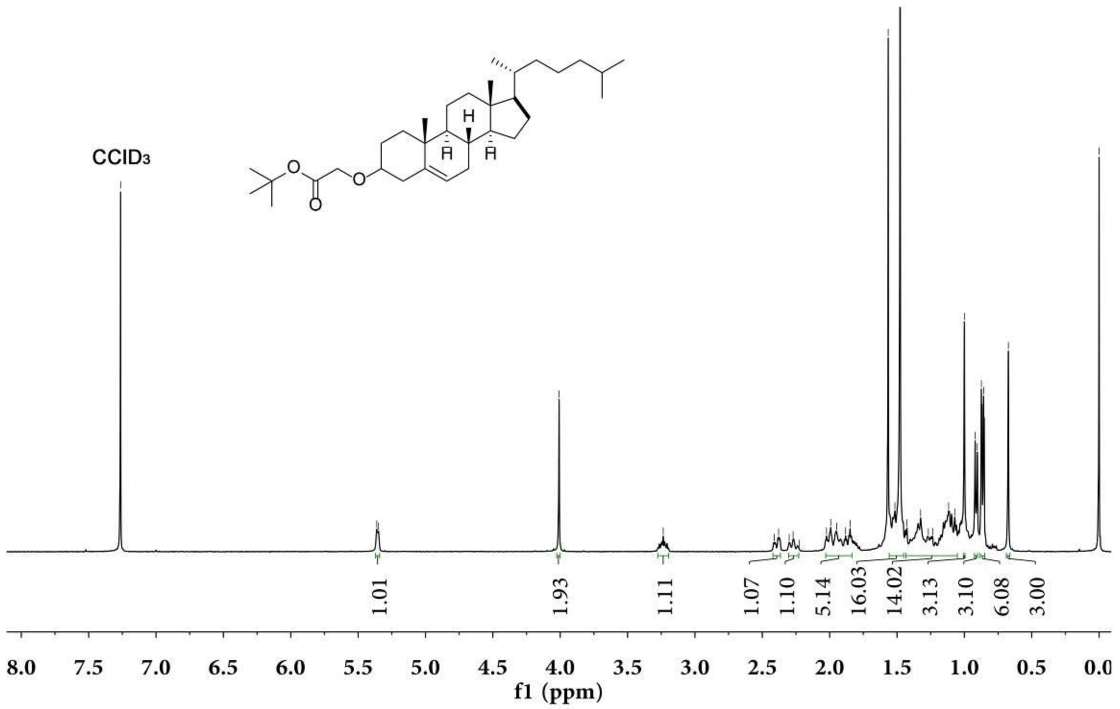

Figure S20. ^{1}H NMR of *tert*-Butyl cholest-5-en-3β-yloxyacetate in $CDCl_3$.

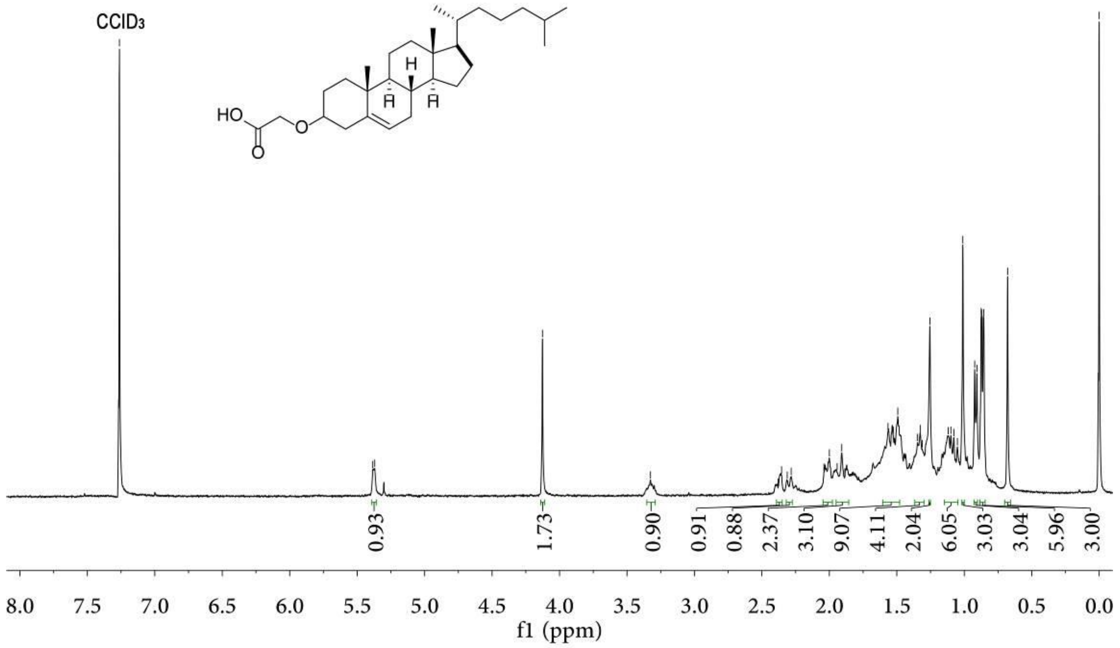

Figure S21. ^{1}H NMR of cholest-5-en-3β-yloxyacetic acid in $CDCl_3$.

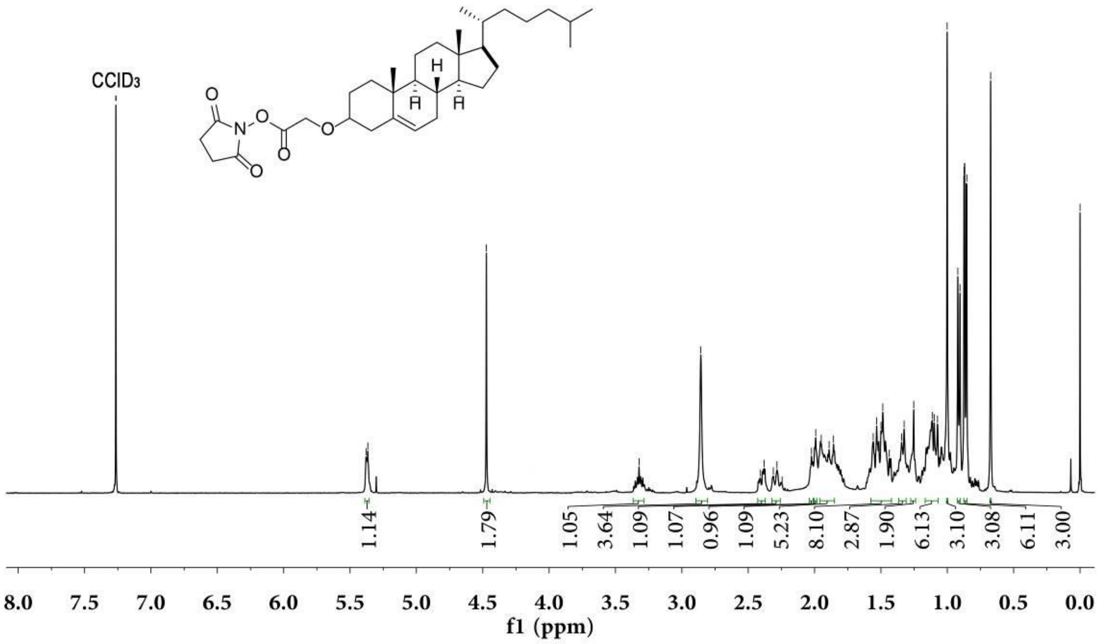

Figure S22. ^{1}H NMR of (2, 5-Dioxopyrrolidin-1-yl) cholest-5-en-3β-yloxyacetic acid in $CDCl_3$.

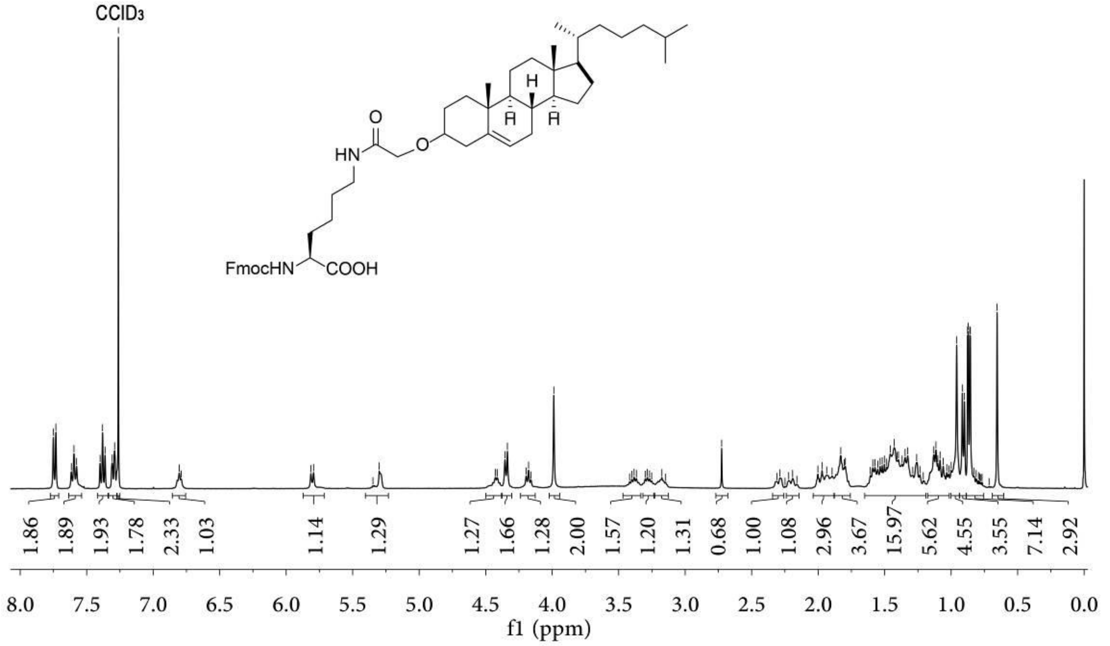

Figure S23. ^{1}H NMR of Fmoc-Lys(cholesterol)-OH in $CDCl_3$.

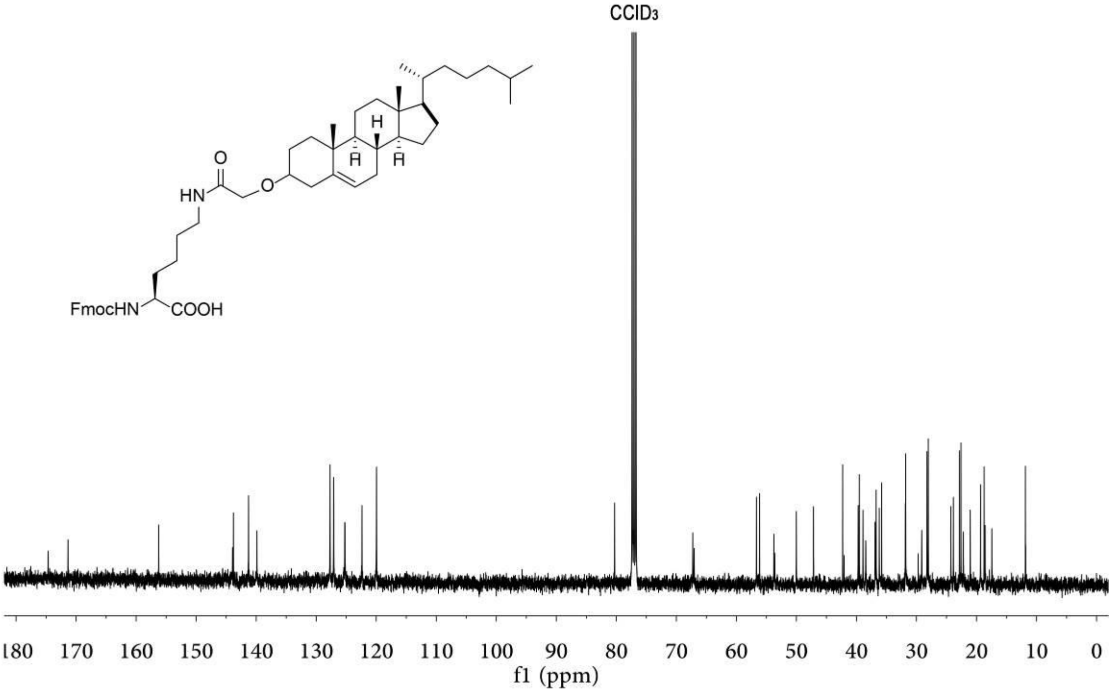

Figure S24. ^{13}C NMR of Fmoc-Lys(Cholesterol)-OH in $CDCl_3$.

Elemental Composition Report Page 1

Single Mass Analysis
Tolerance = 500.0 PPM / DBE: min = -1.5, max = 100.0
Element prediction: Off
Number of isotope peaks used for i-FIT = 3

Monoisotopic Mass, Even Electron Ions
34 formula(e) evaluated with 1 results within limits (up to 1 closest results for each mass)
Elements Used:
C: 0-50 H: 0-71 N: 0-2 O: 0-6 Na: 0-1

JC-WU ECUST institute of Fine Chem 22-Oct-2016 20:31:50
JC-LS-4 224 (2.818) Cm (223:229) 1: TOF MS ES+ 1.19e+003

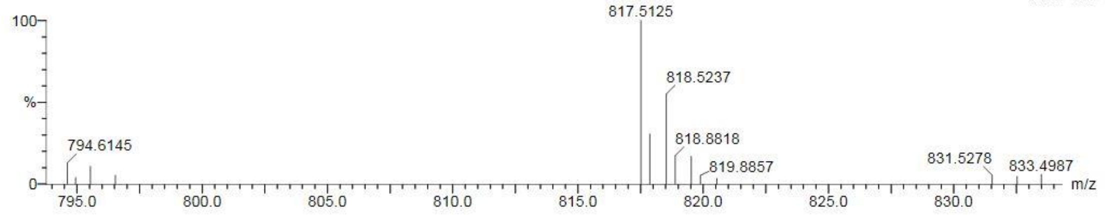

		mDa	PPM	DBE			
Minimum:				-1.5			
Maximum:		300.0	500.0	100.0			
Mass	Calc. Mass	mDa	PPM	DBE	i-FIT	i-FIT (Norm)	Formula
817.5125	817.5132	-0.7	-0.9	16.5	41.7	0.0	C50 H70 N2 O6 Na

Figure S25. HRMS-ESI of Fmoc-Lys(cholesterol)-OH.

23. References:

1. Gao, W.; Xing, LW.; Qu, P.; Tan, TT.; Yang, N.; Li, D.; Chen, HX.; Feng, XJ. *Scientific Reports* **2015**, *5*, 17260.
2. Chen, Y. H.; Yang, J. T.; Chau, K. H. *Biochemistry*, **1974**, *13*, 3350.
3. B.; Jacques, I.; Anilkumar, P.; Gombert, K.; Ducongé, F.; Doris, E. *Nanoscale,* **2013**, *5*, 1955.
